# Symbolic universes between present and future of Europe. First results of the map of European societies' cultural milieu

**DOI:** 10.1371/journal.pone.0189885

**Published:** 2018-01-03

**Authors:** Sergio Salvatore, Viviana Fini, Terri Mannarini, Giuseppe Alessandro Veltri, Evrinomi Avdi, Fiorella Battaglia, Jorge Castro-Tejerina, Enrico Ciavolino, Marco Cremaschi, Irini Kadianaki, Nikita A. Kharlamov, Anna Krasteva, Katrin Kullasepp, Anastassios Matsopoulos, Claudia Meschiari, Piergiorgio Mossi, Polivios Psinas, Rozlyn Redd, Alessia Rochira, Alfonso Santarpia, Gordon Sammut, Jaan Valsiner, Antonella Valmorbida, on behalf of the Re.Cri.Re. Consortium

**Affiliations:** 1 Department of History, Society and Human Studies, Università del Salento, Lecce (LE), Italy; 2 Centro Studi di Psicoterapia e Psicologia della Salute-Istituto Scientifico Biomedico Euro-Mediterraneo (CESP-ISBEM), Mesagne (Brindisi), Italy; 3 School of Media, Communication and Sociology, University of Leicester, Leicester, United Kingdom; 4 School of Psychology, Aristotle University of Thessaloniki, Thessaloniki, Greece; 5 Fakultät für Philosophie, Wissenschaftstheorie und Religionswissenschaft, Ludwig-Maximilians Universitaet München, München, Germany; 6 Departamento de Psicologia Basica 1, Universidad Nacional de Educacion a Distancia, Madrid, Spain; 7 Centre d’Études Européennes (CEE), Sciences Po, Paris, France; 8 Department of Psychology, University of Cyprus, Nicosia, Cyprus; 9 Niels Bohr Professorship Centre for Cultural Psychology, Department of Communication and Psychology, Aalborg University, Aalborg, Denmark; 10 Center for European Refugees Migration and Ethnic Studies (CERMES), Department of Political Sciences, New Bulgarian University, Sofia, Bulgaria; 11 School of Natural Sciences and Health, Tallinn University, Tallinn, Estonia; 12 School of Psychology, University of Crete, Rethimno, Crete, Greece; 13 Department of Architecture, Università RomaTre, Roma, Italy; 14 Laboratoire de Psychologie Clinique, de Psychopathologie et de Psychanalyse (LPCPP), Aix Marseille Universitè, Aix-en-Provence, France; 15 Department of Psychology, University of Malta, Msida, Malta; 16 Segretariat General, European Association for Local Democracy (ALDA), Strasbourg, France; Institut Català de Paleoecologia Humana i Evolució Social (IPHES), SPAIN

## Abstract

This paper reports the framework, method and main findings of an analysis of cultural milieus in 4 European countries (Estonia, Greece, Italy, and UK). The analysis is based on a questionnaire applied to a sample built through a two-step procedure of post-hoc random selection from a broader dataset based on an online survey. Responses to the questionnaire were subjected to multidimensional analysis–a combination of Multiple Correspondence Analysis and Cluster Analysis. We identified 5 symbolic universes, that correspond to basic, embodied, affect-laden, generalized worldviews. People in this study see the world as either a) an *ordered universe;* b) a matter of *interpersonal bond;* c) a *caring society;* d) consisting of a *niche of belongingness;* e) a hostile place *(others’ world)*. These symbolic universes were also interpreted as semiotic capital: they reflect the capacity of a place to foster social and civic development. Moreover, the distribution of the symbolic universes, and therefore social and civic engagement, is demonstrated to be variable across the 4 countries in the analysis. Finally, we develop a retrospective reconstruction of the distribution of symbolic universes as well as the interplay between their current state and past, present and future socio-institutional scenarios.

## Introduction

The countries of the European Union are currently experiencing deep socio-political turbulence that undermines social cohesion as well as national and European institutions. This dynamic manifests itself in different forms–such as fragmentation of social cohesion, ideological and religious radicalization, rise of ultra-right parties, waves of xenophobia and populism, decreased solidarity and partnership among European countries, and political paralysis of European governance. These processes share a commonality: the dramatic radicalization of intergroup conflicts. Increasingly, more people feel that the community they belong to is threatened by turmoil generated by an external enemy. Such feelings proliferate at different levels. The nature and extension of the community group may vary–being either the local community, the Nation, or the primary social group. The identified enemy is also variable on a case-by-case basis–it can be the political caste, migrants, other European countries, Islamic countries, etc. What remains constant is the affect-laden experience of feeling “under the attack from a threatening other”.

These socio-political dynamics in action are generalized and do not necessarily concern specific issues (e.g., the defence of local or national products; the integration of Muslim communities; European fiscal policy). Instead, they gave rise to general discourses about European unity and identity that have spread over different domains of social and institutional life as well as across segments of population. These dynamics, therefore, should be considered as a global cultural phenomenon, namely a process concerning people’s worldviews, their systems of values and their identities. These worldviews describe how people frame their worlds; we argue that they are key in understanding how cultural processes, such as the recent fragmentation of European society, occur.

### Europe. Unity in diversity

In order to comprehend and deal with the current socio-political dynamics in Europe, a deep understanding of the cultural milieu on which European societies are grounded is needed. As intended in this study, the culture milieu is the social arena where people communicate, act, think and experience life and in so doing reproduce and elaborate symbolic universes. The cultural milieu consists of a plurality of symbolic universes, each of them emerging as a particular interpretation of the cultural milieu.

Policies and methods of social intervention needed to address the current socio-institutional crisis should be designed according to the knowledge of what people feel, think and act. Indeed, people are different [1] and react to policies in ways that reflect these differences.

The study seeks to understand and map not only these differences of worldviews and cultural milieus, but also how it is that the cultural milieu in Europe has changed so rapidly from tolerance of others to seemingly sudden outbursts of intolerance. This recent and sudden shift towards intolerance and European instability seems similar to what already happened in Europe prior to the Second World War. For many people, the very idea that quitting the European Union could be a possibility was simply unthinkable just a decade ago. The same could be said with regards to the fact that the leader of a clearly xenophobic party had a chance to become the next French President of the Republic, as well as the fact that a pro-nazi party became one of the most voted for parties in a recent regional election in Germany and Austria. The socio-economic and political dynamics underpinning these kinds of phenomena may have been simmering for decades as consequence of globalization; however, it is somehow astonishing that their effects are converging so rapidly and have become so diffuse, drawing deep rifts in the political, institutional and social spheres.

This paper contributes to the understanding of European societies’ recent fragmentation and instability by showing how this negative trends are closely linked with the state of the cultural milieus, and it shows why we should be paying attention to these perspectives if we want to design efficient policy to counter them. We report results of an ongoing investigation of the cultural dynamics characterizing European societies, entitled Between the representation of the crisis and the crisis of the representation (Re.Cri.Re; www.recrire.eu), which is a three-year study aimed at analysing the cultural impact of the socio-economic crisis of the last decade in European societies and its implications for policy making.

### Framework: Semiotic cultural psychological theory (SPCT) and symbolic universes

The study is based on semiotic cultural psychological theory (SPCT). SPCT integrates relational psychoanalysis [2–4], Dynamic Systems Theory [5–8] and pragmatic semiotics [9, 10] within the more general framework of socio-cultural psychology [11–13].

### Symbolic universes

SCPT conceives mental processes as *ongoing dynamics of sensemaking*. Sensemaking consists of processes of interpretation of the world that shape experience [10, 14]. These processes of interpretation are guided by generalized, affect-laden meanings that are embedded in the cultural milieu and work as basic intuitive assumptions concerning the world as a whole–what it is and how it works–. These intuitive assumptions channel lower generalized meanings, namely specific concepts and opinions concerning facts and objects of the social and physical world, values, and beliefs, attitudes. SCPT adopts the term *symbolic universes* to denote such systems of assumptions.

It is worth adding that SCPT uses such notion in a specific way with respect to how Berger and Luckmann [15] used it–indeed, in the SCPT framework it is meant to highlight two main characteristics of the systems of assumptions:

a) their affective, *pre-semantic* valence–they are used by people in socially suggested directions before being articulated and made linguistically, therefore justified rationally [12];

b) the fact that they envelope the entire field of experience, rather than single parts of it. They function as the *universe of sense* individuals have created for themselves, and in which they are completely embedded. It is in that sense that they are generalized meanings [10].

**Fig 1:**
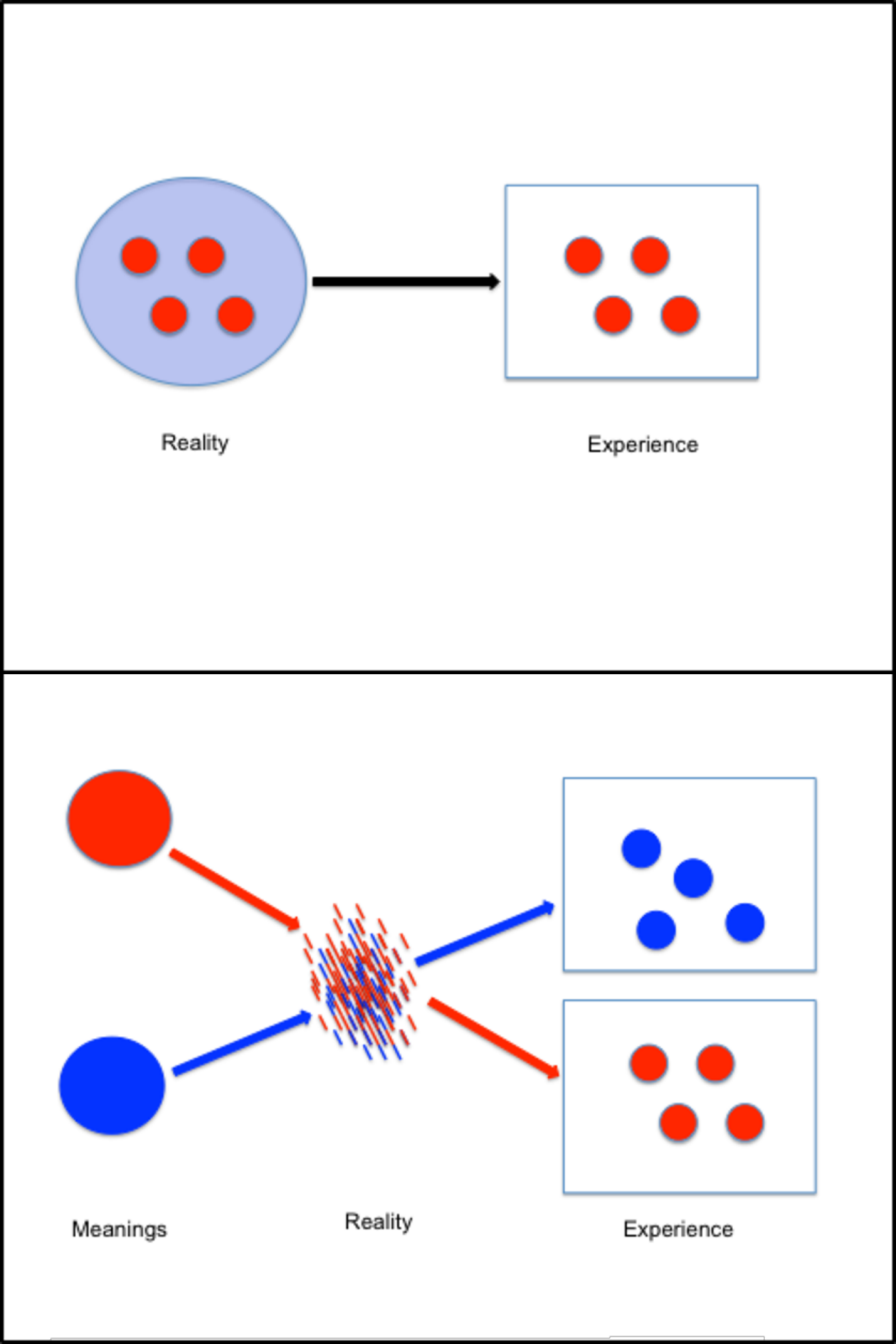
Two views of the relation between reality and experience. A) The view of the experience as a direct function of the reality. B) Semiotic Cultural Psychology Theory’s view. The meaning makes (some elements of realty) pertinent, providing them with a shape. In so doing, they are constituted as contents of experience.

Fig 1 provides a visual description of the dynamics of sensemaking and of the role that symbolic universes play in it. Meaning is not attributed to contents of the world that exist before being interpreted (this view is illustrated by Fig 1A). Rather, sensemaking makes up the reality. Sensemaking does not create the world but shapes the manner of experiencing it (cf. Fig 1B). The person’s sensemaking is guided and shaped by the symbolic universe the sensemaker identifies with.

In sum, a symbolic universe is an affect-laden, pre-semantic meaning working as a basic, generalized assumption that shapes the experience of both the outer (i.e. the social and physical space) and inner environment (i.e., the experience of one’s body and feelings), namely, the embodied image the sensemaker has of oneself and of one’s relation with the world.

### A cultural milieu consists of a plurality of symbolic universes

SCPT conceives of the cultural milieu as an heterogeneous scenario: in every cultural milieu there is variability in the ways of feeling, thinking and behaving. Accordingly, SCPT assumes that any cultural milieu consists of a plurality of symbolic universes. Each symbolic universe emerges as a particular interpretation of the same cultural milieu [14, 16]—where such interpretation consists of emphasizing certain basic dimensions of the experience and de-emphasizing others [17]. Thus, the fact that persons are embedded in the same cultural milieu does not mean that they have the same feelings, ideas and behavioural manifestations. Rather, it means that the variability in their feelings, thoughts and acts reflects (i.e. it is channelled and constrained by) the plurality of the symbolic universes comprising that cultural milieu.

In sum, SCPT conceives of the cultural milieu as the grounds of variability of individual trajectories of sensemaking within a certain society. The cultural milieu is the landscape that defines the movements of feelings, thoughts and actions that are possible in a certain society. Thus, according to SCPT, cultural analysis is not aimed at understanding not only what people share but what makes them differ in their worldviews.

### How to detect the symbolic universes

Due to its embodied nature, a symbolic universe is not directly observable. It can be detected only through abductive reasoning, that is by analysing its effects in terms of how it shapes the sensemaker’s concrete acts of interpretation [10, 18]–in this case study we analyse acts of interpretation provoked by the survey instrument (see below, paragraph Method).

To this end, it is worth taking into account the generalized, affective, pre-semantic nature of the meaning substantiating any symbolic universe. The action associated with a symbolic universe consists of the fact that the sensemaker makes sense of her/his experience in the terms of a pattern of signs–i.e. ideas, attitudes, statements, feelings, habits–that cross over different contexts and objects of experience (e.g. the experience of the place where they live, the micro-social context, the trustworthiness of local services, the vision of the country’s future) in a sufficiently stable and homogeneous way regardless of the semantic linkages among them. Accordingly, any symbolic universe can be identified by means of a procedure of pattern recognition–namely as a pattern of co-occurring signs whose reciprocal association is not (or is only weakly) justified by semantic linkages and therefore can be interpreted as due to the homogenizing and generalizing action of the symbolic universe, namely the symbolic universe’s capacity of activating affective, pre-semantic linkages among signs [19–20].

### The use of symbolic universes for segmenting European societies

Symbolic universes concern the basic meanings substantiating a cultural milieu. They therefore, cannot be used as a construct describing individual psychological characteristics (i.e. as mental models or as personality traits). However, the sensemaker can be characterized in terms of the symbolic universe she/he identifies with *preferentially*. It follows that symbolic universes can be used for both mapping and explaining inter-individual variability in feelings, thoughts and acting expressed by a certain audience. Please, note that here and henceforth we adopt the term *audience* in a broad, general sense, for denoting a given social group–e.g., the people of a European country–that are the target of a policy (for a similar use, see [21]). Accordingly, the term does not imply any view of the target as a passive addressee of the policy. This is so because sensemaking is inherently active, due to the fact that consists of active acts of interpretation performed by people through the mediation of the symbolic universes within which they are embedded. Indeed, on the one hand, the social group can be segmented in terms of the symbolic universes that are active within the cultural milieu that the social group is embedded in; on the other hand, the differences in feeling, thoughts and actions among segments can be explained in terms of the generalized meanings each symbolic universe consists of.

It is beyond the scope of this work to analyse what makes a certain individual identify with a certain symbolic universe and not another, as well as the issue of the extent to which such identification is stable over time. Here we limit ourselves to note that due to its generalized affective characteristic, identification with a symbolic universe tends to be relatively stable over time, even if it can change over the medium-long temporal scale. Moreover, this may depend on a dynamic combination of psychological and biographical factors (e.g. significant relational experiences, life events, exposure to communicative contexts; see Salvatore [10] as well as micro and macro-social conditions (e.g. gender, social class, economic and educational level, occupation, dimension of the family nucleus, religion, social capital) [22, 23].

It is worth noting the peculiarity of segmentation in terms of symbolic universes (henceforth: SCPT segmentation) as opposed to models of audience segmentation prevalent in marketing and socio-political communication. Most of these models adopt socio-demographic parameters and/or characteristics that are specific to the attitude/behaviour the segmentation is aimed at addressing. Just to take a few examples among the very many, Roser-Renouf and colleagues [21] differentiated the US population in 6 audiences, each of them characterized by a specific attitude towards climate change and the propensity to act–or not act–in order to reduce greenhouse gas emissions. The authors did so by means of a survey comprised of 36 variables concerning 4 constructs—global warming beliefs, issue involvement, policy preferences, and behaviours—all of them concerned with the issue at stake (i.e. climate change). With regards to the domain of health interventions, Campo and colleagues [24] based their segmentation on the assumption that “the variables for audience segmentation should be based on the relationship of the variables to the outcome behaviour of interest” (p. 100). Accordingly, they segmented the target population of health interventions aimed at reducing unintended pregnancy among adult women according to parameters concerning the aim of the intervention directly–e.g., perceived risk, fear associated with unintended pregnancy, and age.

By contrast, SCPT segmentation defines the groups in terms of generalized meanings characterizing the global relation between the sensemaker and the world. As consequence, SCPT segmentation is less useful in addressing domain-specific attitudes and behaviours, but it is fully consistent with the goal of understanding and addressing the generalized, trans-domain cultural dynamics characterizing society as a whole.

### Aims

The current paper pursues two objectives:

A) To identify the symbolic universes characterizing a subset of European societies’ cultural milieus and analyse their socio-demographic profile;
B) To segment European societies involved in the analysis according to the symbolic universes identified.

## Method

### Sample and sampling

The study is based on an online survey applied to a convenience sample (henceforth: Sample 0). In order to improve the balance of data and in accordance to the aims of the study, we extracted two sub-samples from Sample 0:

- *Sample 1*, a stratified non-proportional sample (optimal allocation), designed for the identification of the symbolic universes and the study of their socio-demographic profiles (objective A).
- *Sample 2*, a stratified proportional sample (proportional allocation), designed to carry out the audience segmentation (objective B).

Moreover, we tested the stability of the main results by means of a bootstrapping-like procedure–i.e., we compared the output of the same procedure of multidimensional analysis (see sub-paragraph Identification of the symbolic universes) applied on 10 control samples, designed to be equivalent to Sample 1.

In what follows details about the whole sample and the two sub-samples are provided.

### Sample 0

Sample 0 (*N* = 4753) is a non-probability convenience sample, collected by means of a mixture of snowball procedure and specifically designed communicational actions (e.g. presentation of the survey on social networks and in public contexts/events, addressed both to general and *ad hoc* audiences—local administrators, economic operators, academic teachers and students). It comprises respondents from 8 European countries (Cyprus, Estonia, France, Greece, Italy, Malta, Spain, and UK). Approximately 90% of respondents completed an online version of the survey instrument, and about 10% responded to a paper-and-pencil version. In the case of UK, a stratified random sample was considered instead of a non-probability sample. The stratification criteria were gender, age, education and region (i.e., NUTS1 geographical units).

Sample 0 consists of the set of participants involved in the survey from November 3, 2015 to June 6, 2016. The involvement of participants was carried out accordingly to the ethical norms of each country. Participants with more than 25% of unanswered items were excluded. Accordingly, the size of Sample 0 was *N* = 4,753 out of 5,957 persons who completed the survey. Sample 0 is characterized by a higher proportion of women compared to the European population (here and henceforth, source: Eurostat) (62.9% vs. 51.2%) and a lower and more homogeneous age–*M* = 39.58 (*SD* = 16.01) vs. *M* = 41.47 (*SD* = 23.15). Moreover, sample 0 was marked by a higher proportion of lower and higher education levels compared to the European population -lower secondary: 31.7% (European population: 27.5%); upper secondary and post-secondary, non-tertiary 34.7% (European population: 46.6%); tertiary education: 33.6% (European population: 26.0%).

### Sample 1

Sample 1 (*N* = 616) is a stratified, non-proportional quota sample by country, gender and 3 age levels (18–39 yrs; 40–64 yrs; >64 yrs), randomly extracted from sample 0.

The structure of sample 1 meets the criterion of *maximum variability (*also defined as *optimal allocation)*, according to which the sample has to mirror as closely as possible the population’s variability, regardless of the probability associated with states (for a similar procedure see [25, 26]; for a discussion, see [18]). In any population there are patterns of conditions that even if quantitatively marginal, may have an important heuristic value. Such marginal patterns would have a very limited probability of being selected in the case of a representative sample. This is why this sample was extracted using the principle of maximum variability.

The three variables adopted for extracting sample 1 -that is, country of residence, sex and age—were chosen because they were considered the ones with the highest chance of being associated with cultural variability (e.g. [25]).

The extraction was applied separately for each country; *n* = 15 was the designed number of participants for each of the 6 cells (gender*3 levels [18–39 yrs; 40–64 yrs; >64 yrs] age). Indeed, this number was considered the best way of optimizing, on the one hand, the size of the single block and, on the other hand, the need to assure a balanced sample. Countries were included in the analysis if the corresponding subsample presented at least 4 out of 6 cells with *n* > 8.

Sample 1 comprises the same 8 European countries covered by Sample 0. Among these, 4 reached the designed distribution (*n* = 15*6 cells): Estonia (with the exception of one respondent), Greece, Italy, and the UK. In most of the other countries (France, Malta, and Cyprus) the cells that could not be fully accomplished are those concerning the highest age level (cf. Table 1). Taken as a whole, the distribution between age levels is homogeneous for the first two levels (both 38%), however, the third age level is represented with quite a high proportion (24%). Due to its non-proportional structure, Sample 1 is older than the European population (*M* = 46.4 years *vs*. *M* = 41.47). Gender is quite homogeneously distributed between women (52%) and men (48%), which is roughly the same distribution (51.2%) as in the European population. Similarly, the distribution of educational levels is homogeneous—lower secondary or lower levels (i.e. <5 years, 6–9 years and 10–13 years): 33.5%; upper secondary and post-secondary, non-tertiary (14–17 years): 30.8%; tertiary education (>17 years): 35.7% (cf. Table 2). 82.5% of respondents filled the on-line version of the survey with the rest of the sample opting for the paper and pencil version.

**Table 1:** Sample 1. Country*Sex*Age.

*COUNTRY *	*SEX/AGE*						*TOTAL*
	*M/18-39y*	*M/40-64y*	*M/>64y*	*F/18-39y*	*F/40-64y*	*F/>64y*	
*Estonia*	15	15	15	15	15	15	90
% within country	16.70%	16.70%	16.70%	16.70%	16.70%	16.70%	
% within age x gender	12.90%	13.20%	22.70%	12.60%	12.50%	18.50%	14.60%
*Spain*	15	15	2	15	15	1	63
% within country	23.80%	23.80%	3.20%	23.80%	23.80%	1.60%	
% within age x gender	12.90%	13.20%	3.00%	12.60%	12.50%	1.20%	10.20%
*France*	13	9	2	15	15	4	58
% within country	22.40%	15.50%	3.40%	25.90%	25.90%	6.90%	
% within age x gender	11.20%	7.90%	3.00%	12.60%	12.50%	4.90%	9.40%
*Greece*	15	15	15	15	15	15	90
% within country	16.70%	16.70%	16.70%	16.70%	16.70%	16.70%	
% within age x gender	12.90%	13.20%	22.70%	12.60%	12.50%	18.50%	14.60%
*Italy*	15	15	15	15	15	15	90
% within country	16.70%	16.70%	16.70%	16.70%	16.70%	16.70%	
% within age x gender	12.90%	13.20%	22.70%	12.60%	12.50%	18.50%	14.60%
*Cyprus*	13	15	2	14	15	15	74
% within country	17.60%	20.30%	2.70%	18.90%	20.30%	20.30%	
% within age x gender	11.20%	13.20%	3.00%	11.80%	12.50%	18.50%	12.00%
*Malta*	15	15	0	15	15	1	61
% within country	24.60%	24.60%	0.00%	24.60%	24.60%	1.60%	
% within age x gender	12.90%	13.20%	0.00%	12.60%	12.50%	1.20%	9.90%
*United Kingdom*	15	15	15	15	15	15	90
% within country	16.70%	16.70%	16.70%	16.70%	16.70%	16.70%	
% within age x gender	12.90%	13.20%	22.70%	12.60%	12.50%	18.50%	14.60%
*Total*	116	114	66	119	120	81	616
% within Country	18.80%	18.50%	10.70%	19.30%	19.50%	13.10%	

**Table 2:** Sample 1. Education.

*LEVELS*	*N*	*%*
*< 5y*	15	2.8
*6-9y*	67	12.3
*10-13y*	100	18.4
*14-17y*	167	30.8
*> 17 y*	194	35.7
*Missing*	73	
*Total*	616	

### Sample 2

The segmentation is based on the stratified proportional sample (i.e. proportional allocation), focused on the subset of European countries for which enough data were available. In order to optimize the size of each country’s subsample, Sample 2 was designed in accordance with a clustered structure, that is the number of participants for each country’s subsample was defined independently, without taking into account the country’s proportion of population with respect to the European population as a whole.

Sample 2 (*N* = 1,759) retained Sample 1’s 6-cell structure (gender*3 levels [18-39/40-64/>64] age). While Sample 1 was designed to maximize variability, Sample 2 was stratified such that the number of cases in each cell is representative to the corresponding distribution of each country’s population. Due to this design, Sample 2 encompasses 4 countries (Estonia, Greece, Italy, and United Kingdom), those for which enough respondents could be used to create a representative stratification. The Estonian and Greek subsamples’ stratification fitted the corresponding country’s distribution fully. In the case of the UK subsample, the older men’s cell was slightly over-represented (sample: 13% *vs* population: 9.93%). In the case of Italy, the subsample’s relative frequency of older respondents (both men and women) is lower than that of the population (male: 6.89% *vs* 11.20%; women: 4.76% vs 14.88%) (cf. Table 3).

**Table 3:** Sample 2. Country*Sex*Age.

*COUNTRY*	*SEX/AGE*				*TOTAL*		
	*M/18-39y*	*M/40-64y*	*M/>64y*	*F/18-39y*	*F/40-64y*	*F/>64y*	
*Estonia*	64	68	27	61	75	53	348
Sample % within country	18.40%	19.50%	7.80%	17.50%	21.60%	15.20%	
Population % within country	18.48%	19.47%	7.77%	17.42%	21.55%	15.30%	
% within age x gender	19.70%	17.80%	15.30%	19.00%	18.90%	33.10%	
*Greece*	63	75	42	62	81	53	376
Sample % within country	16.64%	19.99%	11.17%	16.39%	21.42%	14.04%	
Population % within country	16.69%	20.05%	11.21%	16.44%	21.50%	14.12%	
% within age x gender	19.40%	19.70%	23.90%	19.30%	20.50%	33.10%	
*Italy*	85	110	31	85	118	21	450
Sample % within country	18.90%	24.40%	6.89%	18.89%	26.22%	4.67%	
Population % within country	15.35%	21.40%	11.20%	14.99%	22.19%	14.88%	
% within age x gender	26.20%	28.90%	17.60%	26.50%	29.80%	13.10%	
*United Kingdom*	113	128	76	113	122	33	585
Sample % within country	19.30%	21.90%	13.00%	19.30%	20.90%	5.60%	
Population % within country	17.67%	22.25%	9.95%	25.13%	20.68%	4.32%	
% within age x gender	12.90%	13.20%	22.70%	12.60%	12.50%	18.50%	
*Total*	325	381	176	321	396	160	1759
% within country	18.50%	21.70%	10.00%	18.20%	22.50%	9.10%	

Table 4 compares the sample’s geographical distribution with that of the corresponding population. The comparison is based on the NUTS1 (Nomenclature of Territorial Units for Statistics-level 1) segmentation (the comparison does not concern Estonia, given that this country is not differentiated at the NUTS1 level):

**Table 4:** Sample 2. Country’s population (>18 years)* NUT1.

* *	*NUT1*	*N*	*Sample (%)*	*Population*
*Greece*	*EL3-Attica*	34	9.0	35.5
	*EL4_Crete and Egean Islands*	43	11.4	25.4
	*EL5_Northern Greece*	207	55.1	10.5
	*EL6-Central Greece*	92	24.5	28.7
* *	*Total*	376	100	100
*Italy*	*ITC-North West Italy*	48	10.7	26.7
	*ITF-South Italy*	128	28.4	23.0
	*ITG-Islands*	7	1.6	11.1
	*ITH-North East Italy*	35	7.8	19.2
	*ITI-Centre*	232	51.6	20.0
	*Total*	450	100	100
*United Kingdom*	*UKC-North East England*	32	5.5	4.1
	*UKD-North West England*	56	9.6	11.0
	*UKE-Yorkshire and the Humber*	55	9.4	8.3
	*UKF-East Midlands*	51	8.7	7.2
	*UKG-West Midlands*	56	9.6	8.7
	*UKH-East of England*	57	9.7	9.3
	*UKI-Great London*	61	10.4	13.1
	*UKJ-South East England*	55	9.4	13.7
	*UKK-South West England*	56	9.6	8.5
	*UKL-Wales*	33	5.6	4.8
	*UKM-Scotland*	56	9.6	8.5
	*UKN-Northern Ireland*	17	2.9	2.8
	Total	585	100	100

Population > 18 years

- The Greek subsample is highly concentred in the Northern Greece (55.1% of the sample *vs* 10.5% of population), whereas it presents a lower proportion of respondents from the other 3 regions—especially Attica (9.0% *vs* 35.5%) and Crete and Aegean Island (14.4% *vs* 25.4%).
- The Italy subsample is highly concentrated in the Centre region (51.6% *vs* 20.0%), whereas it is underrepresented in North West Italy (26.7% *vs* 10.7%), and above all Islands (1.6% *vs* 11.1%).
- The UK subsample approximates the corresponding population’s geographical distribution–the highest difference consists of 2.7 points (Great London: 10.4% *vs* 13.1%)

Regarding education, the two highest levels correspond to more than half of the sample—lower secondary or lower levels (i.e. < 5 years, 6–9 years and 10–13 years): 37.2%; upper secondary and post-secondary, non-tertiary (< 14–17 years): 32.9%; tertiary education (> 17 years): 29.9% (cf. Table 5). 93.8% of respondents filled the on-line version of the survey whilst the rest of the sample used the paper and pencil version.

**Table 5:** Sample 2 Education.

*LEVELS*	*N*	*%*
*< 5y*	50	3.2
*6-9y*	149	9.5
*10-13y*	384	24.5
*14-17y*	516	32.9
*> 17 y*	468	29.9
*Missing*	192	
*Total*	1759	100

### Instrument

The mapping of symbolic universes was based on the VOC (*View of Context*) survey instrument (cf. S1 Text; see [27] too). VOC is a 68-item questionnaire that assesses how people represent affect-laden, significant aspects of their life and context. The questionnaire is composed of 2 parts: a) the first part concerns views/evaluations of aspects of the place where the respondent lives (e.g., the reliability of agencies and services; quality of life in the coming years); b) the second part concerns views/evaluation of aspects concerning the broader social context and life in general (e.g., system of values; people’s capacity for change; what constitutes success in life; what behaviour depends on). The questionnaire further integrates a set of variables aimed at collecting information on the respondents’ background and the social context they are part of (e. g., socio-demographic characteristics; civil status; size of the family nucleus; place of birth and living; self-evaluation of current health; involvement in volunteer community activities).

VOC is the revised version of a family of questionnaires aimed at analysing the cultural milieu in terms of latent dimensions of sensemaking. Previous versions of the questionnaire have been used for the last 20 years with the aim of analysing the cultural milieu characterizing specific domains of activity (school: [27, 28]; higher education: [17]; organizations: [29]; health: [30]; local community: [31]; local development: [32, 33]; as well as the cultural frame of the representation of social objects -e.g., the profession of psychologist: [25]; urban mobility: [34]; risks in the workplace: [35]; gambling: [26]. All these analyses were performed in Italy. Compared to these previous versions, VOC is shorter and more generalized, focusing on the analysis of how the context–in the sense of experience of the world as a whole–is interpreted. Previous studies conducted on Italian versions of the questionnaire have shown a satisfactory construct validity [25, 31] as well as a satisfactory level of inner consistency (Chronbach’s alpha was .74, cf. [17]).

The language versions of the questionnaire (one for each country involved in the survey) were uploaded on a purpose built webpage, which was accessible both directly (www.okokok.info) and through the webpage of the Re.Cri.Re project (www.recrire.eu). The questionnaire items were organized in one of the two following ways: (a) some items are associated with a four-point Likert scale without intermediate alternatives, purposely chosen as a way of ‘forcing’ the responses towards oppositional modes of response; (b) other items consist of a question associated with alternative, contrasting responses among which the respondent is asked to choose. These items were constructed on the grounds of a methodology integrating psychoanalytic and psycho-cultural standpoints [4, 18, 28, 29, 36] aimed at detecting the oppositional structures underpinning modes of interpreting reality. According to this methodology, items are aimed at facilitating the expression of perceptions/opinions/judgments concerning the micro- and macro-social environment (e.g. evaluation of the place where the person lives; level of trustworthiness of social structures), and in so doing to trigger the activation of generalized meanings.

Four characteristics of the items contribute to this purpose. Firstly, items spread over a plurality of levels and objects of experience (e.g., institutions; quality of life; sense of empowerment; future; rules; interpersonal bonds). Secondly, most of the items are formulated with generic reference (e.g. your future; your life). This is so because when a person is asked to connote an object, the more the object is characterized by specific characteristics the more such characteristics will constrain the way by which it is interpreted. On the other hand, the less specifically the object is defined, the greater the probability that it will work as a projective stimulus triggering emotional forms of connotation. Thirdly, items are designed to go beyond the mere description of states of things. Rather, most items are invitations to assume a position with respect to pressing issues, such as identity-sensitive matters, which are open to contrasting ideological and value-laden options. Fourthly, items are associated to response modes that force the respondent further to take a stance with respect to contrasting positions. This makes the structure of the response isomorphic to the oppositional structure of the dimensions of sense that we intend to detect [20, 31].

## Data analysis

### Identification of the symbolic universes

Symbolic universes were identified by means of a procedure of Cluster Analysis (CA)–hierarchical classification method–aimed at identifying the response profiles associated with different groups of individuals. The CA was based on a Multiple Correspondence Analysis (MCA) of Sample 1 responses to the VOC. Sample 1 respondents contributed to the variance of the MCA as active individuals, and Sample 2 respondents were supplemental individuals who did not contribute to the analysis, but who were mapped onto the MCA space based on Sample 1 respondents (see below, sub-paragraph: Audience segmentation).

Multiple Correspondence Analysis is an extension of correspondence analysis aimed at detecting patterns of association among several categorical variables. Accordingly, it can be conceived of as a generalization of principal component analysis applied on categorical rather than quantitative variables [37] (for a discussion as to the consistency of the MCA with the interpretation of symbolic universes in terms of generalized, affect-laden meaning, see [18].)

MCA and CA were carried out by means of the package SPAD.

The identification of profiles was based on the criterion of maintaining the maximum similarity between the response profiles grouped in the same cluster and the maximum differentiation in the response profiles grouped in different clusters. In our case the similarity/dissimilarity criteria were given by the main factorial dimensions extracted by a previous MCA applied on the same dataset. MCA was adopted because of the metric characteristics of the items (categorical and ordinal scales).

The choice of the optimal partition was driven by the aim of obtaining the highest number of clusters whose further segmentation: a) would not increase the Inter-class inertia/Total inertia ratio greatly; and/or b) would produce clusters with low face validity (i.e., profiles that were low in consistence and hence hard to be interpreted); and/or c) would generate cluster(s) with low frequency (< 5%). It is worth observing that the adoption of the maximum number of clusters (within the constraints of conditions a, b, and c) as optimization criterion was consistent with the purpose of the analysis, which intended to detect the areas of cultural specificity within the cultural milieu. Accordingly, the more the symbolic universes, the more the capacity of the map to provide a valid picture of the cultural milieu, also of its parts that could be marginal from a quantitative standpoint, yet relevant in accordance to a qualitative, strategic point of view (e.g. as potential drivers of innovation) (see above, the criterion of *maximum variability*).

Each cluster produced by the Cluster Analysis consists of a specific profile of individual responses, namely a pattern of responses that tends to co-occur redundantly across the sample, even if they have no semantic and/or functional linkage with each other. Thus, on the grounds of the methodological framework adopted, each cluster was interpreted as the marker of a symbolic universe.

As a complementary output, CA attributed each respondent (both active [Sample 1] and supplemental [Sample 2] individuals) to the most similar cluster. This classification was used for the socio-demographic profiling of the segments and the following audience segmentation (see below, sub-paragraph Audience segmentation).

As to the socio-demographic description, we carried out the following analyses.

A) A univariate analysis with cluster membership as dependent variable and Age as independent variable.
B) A multinomial regression model, with cluster membership as dependent variable and a set of socio-demographic characteristics (Sex, Education, Self-assessment of one’s state of health [henceforth: Health], Size of familiar nucleus [henceforth: Nucleus], Occupation, Civil Status [more specifically, we focused on the following categories of status: Married, Parent, Separated, Widowed, Living with family of origin] and Volunteer activity) as independent variables.
C) Finally, in order to provide a more detailed profile of the symbolic universes, the distribution over the clusters of each socio-demographic characteristic was analysed by means of a chi-square test. These tests were performed only for the socio-demographic characteristics that proved to have significant effect (threshold: p<0.01) in the multinomial model.

Univariate, multinomial and chi-square analyses were performed by means of the SPSS package

### Reliability of the symbolic universes

In order to test the aspect of the reliability concerning the independence of findings from sampling procedures, we adopted a bootstrapping-like logic. More specifically, we randomly extracted 10 control samples from Sample 0 –each control sample was designed as corresponding to Sample 1 with regards to numerousness and 6-cell structure. Then, we repeated the same procedure of multidimensional analysis described above (MCA and CA) on each of these samples. Finally, the output of the main analysis (i.e. the response profiles obtained from Cluster Analysis) was compared with the corresponding outputs of each control sample. More particularly, for each cluster extracted from the main Cluster Analysis, we calculated the percentage of coverage of the list of items characterizing it and the list of items characterizing the corresponding cluster extracted from each control subsample.

### Audience segmentation

With regards to audience segmentation, the Sample 2 participants were classified in accordance to the cluster obtained from previous CA. To this end, as detailed (see sub-paragraph Identification of the symbolic universes), Sample 2 respondents were introduced in the previous CA as “supplemental individuals”, that is, individuals who did not actively contribute to the formation of clusters but who were associated to them once these were formed. The chi-square test was used to compare the distribution of the segments among the countries.

## Results

### Cluster analysis

Cluster Analysis used the factorial dimensions extracted by the Multiple Correspondence Analysis (number of factor dimensions extracted: 109) as similarity criteria (S1 Table outlines the 3 main factorial dimensions, with terms of the VOC modalities’ coordinates on them. The partition in five clusters was chosen as the optimal solution of Cluster Analysis (Inter-class inertia/Total inertia: 0.203/0.601 = 0.337). Further differentiation did not greatly increase the inter-class/total inertia ratio (e.g. ratio corresponding to 6 Clusters: 0.36), whereas it reduced face validity, that is, it created marginal (*N* < 5%) and/or partitions lacking specific meaning. Table 6 reports the descriptions of the response profiles characterizing the 5 clusters below (the alphanumeric string corresponds to the item’s id, as reported in the table). S1 and S2 Figs report the position of the clusters on the factorial space defined by the main 3 factorial dimensions.

**Table 6:** Cluster analysis output.

*ID*	*Items*	*Modalities*	*%* *modal*.*/ class*	*%* *class/* *modal*.	*Test Values*	*p (0*.*)*	*F*	*% modal*.*/* *sample*
CLUSTER 1								
C1.1	AGREEMENT/DISAGREEMENT-It is useless to bustle, since you cannot affect	strongly disagree	62.38	45.99	9.64	000	137	22.24
C1.2	AGREEMENT/DISAGREEMENT-People are unable to change	strongly disagree	59.41	45.45	9.21	000	132	21.43
C1.3	TO SUCCEED IN LIFE-Forming alliances with stronger people	not at all	42.57	53.75	8.35	000	80	12.99
C1.4	AGREEMENT/DISAGREEMENT-My life is controlled by accidental happenings	strongly disagree	42.57	48.86	7.77	000	88	14.29
C1.5	TO SUCCEED IN LIFE-Adjusting to the main trends	not at all	26.73	60	6.83	000	45	7.31
C1.6	AGREEMENT/DISAGREEMENT-My life is chiefly controlled by powerful others	strongly disagree	48.51	36.30	6.47	000	135	21.92
C1.7	TO SUCCEED IN LIFE-Having a few scruples	not at all	53.47	33.96	6.46	000	159	25.81
C1.8	AGREEMENT/DISAGREEMENT-Those who succeed in the life has luck on their side	strongly disagree	27.72	49.12	5.99	000	57	9.25
C1.9	TO SUCCEED IN LIFE-Sharing	very	63.37	27.59	5.63	000	232	37.66
C1.10	AGREEMENT/DISAGREEMENT-My life is determined by my own actions	strongly agree	50.50	26.29	4.26	000	194	31.49
C1.11	AGREEMENT/DISAGREEMENT-It is not possible at all to make any provision	strongly disagree	27.72	31.82	3.80	001	88	14.29
C1.12	AGREEMENT/DISAGREEMENT-Sometimes to break the rules to help one’s loved	strongly disagree	16.83	40.48	3.74	001	42	6.82
C1.13	WELLBEING IS-Safety	No	43.56	25.43	3.56	002	173	28.08
C1.14	AGREEMENT/DISAGREEMENT-Immigrants are a source of cultural enrichment	strongly agree	24.75	30.49	3.33	004	82	13.31
C1.15	TO SUCCEED IN LIFE-Following rules	not at all	11.88	42.86	3.23	006	28	4.55
C1.16	AGREEMENT/DISAGREEMENT-There's little use in writing to public officials	strongly disagree	14.85	37.50	3.20	007	40	6.49
C1.17	BEHAVIOUR DEPENDS ON-Economic interest	No	76.24	19.79	2.94	017	389	63.15
C1.18	AGREEMENT/DISAGREEMENT-It's hardly fair to bring children into the world	strongly disagree	42.57	23.12	2.79	027	186	30.19
C1.19	AGREEMENT/DISAGREEMENT-Nowadays a person has to live pretty much for today	strongly disagree	22.77	28.05	2.76	029	82	13.31
C1.20	FUTURE WILL BE-	far better	15.84	31.37	2.64	042	51	8.28
C1.21	CURRENT LIFE	Much better	13.86	32.56	2.57	051	43	6.98
C1.22	BEHAVIOUR DEPENDS ON-The need to defend one’s reputation	No	96.04	17.73	2.55	053	547	88.80
C1.23	BEHAVIOUR DEPENDS ON-Shared values	Yes	35.64	23.38	2.51	060	154	25
C1.24	AGREEMENT/DISAGREEMENT-A person doesn't really know whom he can count on	strongly disagree	11.88	34.29	2.51	060	35	5.68
C1.25	WELLBEING IS-Not being ill	No	63.37	20.13	2.49	065	318	51.62
C1.26	RELIABILITY-Police	not very	36.63	22.70	2.36	091	163	26.46
CLUSTER 2								
C2.1	AGREEMENT/DISAGREEMENT-It is useless to bustle, since you cannot affect	quite disagree	73.29	43.70	8.73	000	270	43.83
C2.2	AGREEMENT/DISAGREEMENT-It is not possible at all to make any provision	quite disagree	62.73	48.10	8.67	000	210	34.09
C2.3	AGREEMENT/DISAGREEMENT-A person doesn't really know whom he can count on	quite disagree	56.52	50.56	8.51	000	180	29.22
C2.4	AGREEMENT/DISAGREEMENT- The lot of the average man is getting worse	quite disagree	52.80	52.15	8.40	000	163	26.46
C2.5	FUTURE WILL BE-	a little better	78.88	39.94	8.16	000	318	51.62
C2.6	AGREEMENT/DISAGREEMENT-My life is determined by my own actions	quite agree	77.64	36.34	6.55	000	344	55.84
C2.7	WELLBEING IS-Not suffering	No	80.75	34.67	6.12	000	375	60.88
C2.8	RELIABILITY-Public Administration	quite	62.73	38.26	5.82	000	264	42.86
C2.9	AGREEMENT/DISAGREEMENT-Those who succeed in the life has luck on their side	quite disagree	57.14	39.66	5.78	000	232	37.66
C2.10	AGREEMENT/DISAGREEMENT-My life is chiefly controlled by powerful others	quite disagree	57.14	39.32	5.67	000	234	37.99
C2.11	CURRENT LIFE	Quite better	49.69	41.45	5.63	000	193	31.33
C2.12	TO SUCCEED IN LIFE-Having a few scruples	not very	51.55	40.29	5.48	000	206	33.44
C2.13	RELIABILITY-Health care services	quite	73.29	34.71	5.37	000	340	55.19
C2.14	AGREEMENT/DISAGREEMENT-Nowadays a person has to live pretty much for today	quite disagree	52.17	39.25	5.23	000	214	34.74
C2.15	AGREEMENT/DISAGREEMENT-There's little use in writing to public officials	quite disagree	39.13	43.45	5.15	000	145	23.54
C2.16	TO SUCCEED IN LIFE-Forming alliances with stronger people	quite	56.52	37.60	5.08	000	242	39.29
C2.17	HOW YOU WILL LIVE IN THE PLACE YOU LIVE IN NEXT 5 Y-	quite better	39.75	42.11	4.91	000	152	24.68
C2.18	RELIABILITY-Police	quite	72.05	33.92	4.89	000	342	55.52
C2.19	WELLBEING IS-Not being ill	No	68.32	34.59	4.89	000	318	51.62
C2.20	AGREEMENT/DISAGREEMENT-My life is controlled by accidental happenings	quite disagree	65.84	34.64	4.71	000	306	49.68
C2.21	AGREEMENT/DISAGREEMENT-People are unable to change	quite disagree	56.52	35.69	4.42	000	255	41.40
C2.22	AGREEMENT/DISAGREEMENT-Immigrants are a source of cultural enrichment	quite agree	54.66	35.77	4.31	000	246	39.94
C2.23	WELLBEING IS-Fulfilment	Yes	75.16	32.10	4.22	000	377	61.20
C2.24	WELLBEING IS-Adaptability	Yes	58.39	34.06	3.93	000	276	44.81
C2.25	AGREEMENT/DISAGREEMENT-It's hardly fair to bring children into the world	quite disagree	50.93	35.34	3.91	000	232	37.66
C2.26	RELIABILITY-Companies	quite	72.67	31.62	3.76	001	370	60.06
C2.27	TO SUCCEED IN LIFE-Adjusting to the main trends	quite	55.28	33.46	3.50	002	266	43.18
C2.28	AGREEMENT/DISAGREEMENT-Sometimes to break the rules to help one’s loved	quite disagree	32.92	37.06	3.22	007	143	23.21
C2.29	TO SUCCEED IN LIFE-Sharing	quite	50.93	32.28	2.80	025	254	41.23
C2.30	BEHAVIOUR DEPENDS ON-Shared values	Yes	32.92	34.42	2.56	053	154	25
C2.31	RELIABILITY-Public transport	quite	60.87	30.63	2.55	054	320	51.95
C2.32	BEHAVIOUR DEPENDS ON-The emotions	Yes	54.04	31.30	2.55	054	278	45.13
CLUSTER 3								
C3.1	RELIABILITY-Public Administration	very	46.38	84.21	10.94	000	38	6.17
C3.2	RELIABILITY-Police	very	59.42	61.19	10.87	000	67	10.88
C3.3	RELIABILITY-Health care services	very	68.12	48.45	10.61	000	97	15.75
C3.4	RELIABILITY-Companies	very	42.03	80.56	10.08	000	36	5.84
C3.5	RELIABILITY-Schools	very	69.57	37.21	9.28	000	129	20.94
C3.6	RELIABILITY-Public transport	very	46.38	39.51	7.25	000	81	13.15
C3.7	AGREEMENT/DISAGREEMENT- The lot of the average man is getting worse	strongly disagree	17.39	60	5.21	000	20	3.25
C3.8	AGREEMENT/DISAGREEMENT-A person doesn't really know whom he can count on	strongly disagree	20.29	40	4.43	000	35	5.68
C3.9	FUTURE WILL BE-	a little better	69.57	15.09	3.07	011	318	51.62
C3.10	AGREEMENT/DISAGREEMENT-It is not possible at all to make any provision	strongly disagree	27.54	21.59	2.94	016	88	14.29
C3.11	TO SUCCEED IN LIFE-Following rules	very	33.33	18.55	2.62	044	124	20.13
C3.12	AGREEMENT/DISAGREEMENT-There's little use in writing to public officials	quite disagree	36.23	17.24	2.40	081	145	23.54
C3.13	AGREEMENT/DISAGREEMENT-It's hardly fair to bring children into the world	strongly disagree	43.48	16.13	2.36	092	186	30.19
CLUSTER 4								
C4.1	AGREEMENT/DISAGREEMENT-It is useless to bustle, since you cannot affect	quite agree	46.36	70.83	9.81	000	144	23.38
C4.2	AGREEMENT/DISAGREEMENT-A person doesn't really know whom he can count on	quite agree	70.91	53.61	8.78	000	291	47.24
C4.3	FUTURE WILL BE-	a little worse	45.91	59.41	7.39	000	170	27.60
C4.4	AGREEMENT/DISAGREEMENT- The lot of the average man is getting worse	quite agree	62.73	50	6.60	000	276	44.81
C4.5	AGREEMENT/DISAGREEMENT-People are unable to change	quite agree	42.27	56.36	6.29	000	165	26.79
C4.6	AGREEMENT/DISAGREEMENT-Those who succeed in the life has luck on their side	quite agree	57.73	50.40	6.23	000	252	40.91
C4.7	AGREEMENT/DISAGREEMENT-My life is chiefly controlled by powerful others	quite agree	47.73	53.57	6.17	000	196	31.82
C4.8	AGREEMENT/DISAGREEMENT-It's hardly fair to bring children into the world	quite agree	35.91	58.09	5.96	000	136	22.08
C4.9	AGREEMENT/DISAGREEMENT-It is not possible at all to make any provision	quite agree	46.82	50.99	5.39	000	202	32.79
C4.10	AGREEMENT/DISAGREEMENT-My life is controlled by accidental happenings	quite agree	44.09	51.87	5.38	000	187	30.36
C4.11	RELIABILITY-Public Administration	not very	54.09	47.79	5.05	000	249	40.42
C4.12	RELIABILITY-Health care services	not very	33.64	53.62	4.81	000	138	22.40
C4.13	AGREEMENT/DISAGREEMENT-There's little use in writing to public officials	quite agree	59.55	45.80	4.79	000	286	46.43
C4.14	AGREEMENT/DISAGREEMENT-Sometimes to break the rules to help one’s loved	quite agree	65.45	44.44	4.71	000	324	52.60
C4.15	AGREEMENT/DISAGREEMENT-Nowadays a person has to live pretty much for today	quite agree	49.09	46.55	4.26	000	232	37.66
C4.16	WELLBEING IS-Not suffering	Yes	48.64	44.58	3.57	002	240	38.96
C4.17	AGREEMENT/DISAGREEMENT-Immigrants are a source of cultural enrichment	quite disagree	35	46.95	3.38	004	164	26.62
C4.18	BEHAVIOUR DEPENDS ON-Shared values	No	82.73	39.39	3.26	006	462	75
C4.19	BEHAVIOUR DEPENDS ON-The need to defend one’s reputation	Yes	16.82	53.62	3.10	010	69	11.20
C4.20	RELIABILITY-Police	not very	34.09	46.01	3.08	010	163	26.46
C4.21	CURRENT LIFE	Neither worse nor be	39.09	44.79	3.05	011	192	31.17
C4.22	HOW YOU WILL LIVE IN THE PLACE YOU LIVE IN NEXT 5 Y-	quite worse	18.64	51.90	3.04	012	79	12.82
C4.23	WELLBEING IS-Not being ill	Yes	55.91	41.41	2.77	028	297	48.21
C4.24	BEHAVIOUR DEPENDS ON-Economic interest	Yes	44.09	42.73	2.68	037	227	36.85
C4.25	CURRENT LIFE	Quite worse	20.91	48.42	2.66	039	95	15.42
C4.26	TO SUCCEED IN LIFE-Forming alliances with stronger people	very	21.82	47.06	2.48	066	102	16.56
C4.27	RELIABILITY-Schools	quite	67.27	39.68	2.47	068	373	60.55
C4.28	TO SUCCEED IN LIFE-Understanding the world	quite	49.55	41.29	2.41	079	264	42.86
C4.29	TO SUCCEED IN LIFE-Having a few scruples	quite	32.27	43.56	2.33	0.01	163	26.46
CLUSTER 5								
C5.1	AGREEMENT/DISAGREEMENT-A person doesn't really know whom he can count on	strongly agree	76.92	45.87	11.25	000	109	17.69
C5.2	FUTURE WILL BE-	far worse	61.54	58.82	10.80	000	68	11.04
C5.3	AGREEMENT/DISAGREEMENT-It is useless to bustle, since you cannot affect	strongly agree	58.46	58.46	10.41	000	65	10.55
C5.4	AGREEMENT/DISAGREEMENT- The lot of the average man is getting worse	strongly agree	80	33.33	9.77	000	156	25.32
C5.5	AGREEMENT/DISAGREEMENT-People are unable to change	strongly agree	53.85	56.45	9.70	000	62	10.06
C5.6	AGREEMENT/DISAGREEMENT-My life is chiefly controlled by powerful others	strongly agree	44.62	60.42	8.92	000	48	7.79
C5.7	AGREEMENT/DISAGREEMENT-It is not possible at all to make any provision	strongly agree	64.62	36.84	8.72	000	114	18.51
C5.8	AGREEMENT/DISAGREEMENT-It's hardly fair to bring children into the world	strongly agree	47.69	51.67	8.58	000	60	9.74
C5.9	AGREEMENT/DISAGREEMENT-There's little use in writing to public officials	strongly agree	69.23	31.03	8.24	000	145	23.54
C5.10	AGREEMENT/DISAGREEMENT-Those who succeed in the life has luck on their side	strongly agree	47.69	44.29	7.90	000	70	11.36
C5.11	RELIABILITY-Police	not at all	35.38	57.50	7.57	000	40	6.49
C5.12	HOW YOU WILL LIVE IN THE PLACE YOU LIVE IN NEXT 5 Y-	much worse	30.77	66.67	7.53	000	30	4.87
C5.13	AGREEMENT/DISAGREEMENT-Sometimes to break the rules to help one’s loved	strongly agree	55.38	34.62	7.50	000	104	16.88
C5.14	RELIABILITY-Public Administration	not at all	41.54	42.86	7.11	000	63	10.23
C5.15	AGREEMENT/DISAGREEMENT-Nowadays a person has to live pretty much for today	strongly agree	46.15	34.88	6.67	000	86	13.96
C5.16	RELIABILITY-Health care services	not at all	30.77	50	6.48	000	40	6.49
C5.17	AGREEMENT/DISAGREEMENT-Immigrants are a source of cultural enrichment	strongly disagree	52.31	27.87	6.13	000	122	19.81
C5.18	AGREEMENT/DISAGREEMENT-My life is controlled by accidental happenings	strongly agree	24.62	53.33	5.89	000	30	4.87
C5.19	RELIABILITY-Companies	not at all	26.15	45.95	5.61	000	37	6.01
C5.20	CURRENT LIFE	Much worse	16.92	52.38	4.72	000	21	3.41
C5.21	TO SUCCEED IN LIFE-Forming alliances with stronger people	very	40	25.49	4.71	000	102	16.56
C5.22	CURRENT LIFE	Quite worse	36.92	25.26	4.43	000	95	15.42
C5.23	RELIABILITY-Schools	not very	36.92	24.74	4.33	000	97	15.75
C5.24	WELLBEING IS-Not being ill	Yes	73.85	16.16	4.30	000	297	48.21
C5.25	WELLBEING IS-Not suffering	Yes	64.62	17.50	4.29	000	240	38.96
C5.26	TO SUCCEED IN LIFE-Having a few scruples	very	29.23	25.33	3.82	001	75	12.18
C5.27	WELLBEING IS-Fulfilment	No	60	16.39	3.55	002	238	38.64
C5.28	RELIABILITY-Schools	not at all	10.77	50	3.54	002	14	2.27
C5.29	TO SUCCEED IN LIFE-Following rules	very	38.46	20.16	3.50	002	124	20.13
C5.30	CURRENT HEALTH	Bad	16.92	29.73	3.18	007	37	6.01
C5.31	AGREEMENT/DISAGREEMENT-My life is determined by my own actions	strongly agree	49.23	16.49	3.03	012	194	31.49
C5.32	TO SUCCEED IN LIFE-Acquiring knowledge	not very	12.31	34.78	3	014	23	3.73
C5.33	BEHAVIOUR DEPENDS ON-Economic interest	Yes	53.85	15.42	2.82	024	227	36.85
C5.34	TO SUCCEED IN LIFE-Following rules	not at all	12.31	28.57	2.54	056	28	4.55
C5.35	RELIABILITY-Public transport	not very	41.54	16.07	2.51	061	168	27.27
C5.36	TO SUCCEED IN LIFE-Adjusting to the main trends	very	29.23	18.27	2.50	062	104	16.88
C5.37	WELLBEING IS-Adaptability	No	69.23	13.27	2.33	0.01	339	55.03

Items included ifa bove the threshold p>.01

In what follows, a brief description of the 5 response profiles is provided (cf. Table 7; the alphanumeric codes correspond to the items reported in Table 6). Each of these is associated with its interpretation in terms of a symbolic universe, which we have included here for the sake of clarity. The label of the symbolic universes has been chosen to represent their core meaning.

**Table 7:** Description of clusters and their interpretation in terms of symbolic universes.

*Response profile*	*Interpretation*
Cluster 1 The profile is characterized by extreme responses. Faith in people [C1.24;], sense of agency [C1.4; C1.8] and possibility of contributing to make things better [C1.1; C1.19]; rejection of power [C1.7], opportunism (C1.3) and conformism [C1.5; C1.22]; solidarity, sharing [C1.9; C1.23], commitment (C1.2), valorisation of otherness [C1.14]; centrality of values in life. People do not act according to economic interest [C1.17]. Rightness [C1.7], morality [C1.12], efficacy [C1.10] are the values of reference; confidence about the future [C1.20].	Symbolic Universe 1. *Ordered universe* Cluster 1 is characterized by two relevant facets–on the one hand, a generalized positive attitude toward the world (institutions and services, the people, the place where one lives, the country, the future) which is considered trustworthy, receptive of the efforts to engage with and to improve it. On the other hand, there is identification with transcendent values and ideals (e.g. justice, morality, solidarity; rejection of opportunism, conformism and power) that foster commitment to making things better—where such commitment is meant as a value in itself: the way of making life meaningful, rather than of pursing material interests. The combination of these two facets outlines what we interpret as the basic assumption substantiating this symbolic universe: faith in the inherent ethical order of the world. Rightness, morality and efficacy go together, what is just is also efficacious in rendering things better, because the universe follows its own harmonious design. Behaviour has to conform to and reflect such universal order and in so doing one can trust in being on the right side of the history.
Cluster 2 The experience of being part of vital interpersonal bonds [C2.32]–to love, to share [C2.29; C2.30], to trust [C2.3]—is what makes life meaningful and fulfilling. Success in life depends on them [C2.1; C2.2]; wellbeing is a matter of adaptability [C2.24] and fulfilment [C2.23] at the same time; conformism is considered a route to success in life-main trends [C2.16]. Moderate sense of agency [C2.6; C2.9] and trust in the possibility of making things better [C2.1]; moderate optimism in the future [C2.5] and in agencies [C2.8; C2.13; C2.18; C2.26; C2.31]; openness to diversity [C2.22].	Symbolic Universe 2. *Interpersonal bond* Cluster 2 comprises a group of responses detecting a positive, optimistic vision of the world, as a place that is meaningful and fulfilling. On the other hand, the world these responses refer to is not the universalistic one of the previous symbolic universe; rather it is the vital world of interpersonal, emotional bonds. To be part of such a world is an end in itself: sacrifices (in terms of adaptability and conformism) are needed for it and are repaid in terms of safety and fulfilment, as well as in promoting a moderate sense of agency, trust and openness to novelty. The verse of the famous song–*all I need is love–*depicts the basic assumption this symbolic universe consists of.
Cluster 3 Full trust in society—its agencies, and institutions [C3.1; C3.2; C3.3; C3.4; C3.5; C3.6; C3.8]. Generalized feelings of confidence with life, sense of agency [C3.10; C3.13] as well as the expectation that the world is going well and will do so in future [C3.7; C3.9]. What one has to do is to respect the rules [C3.11] and highlight one’s needs and demands to those whose role it is to respond to them [C3.12].	Symbolic Universe 3. *Caring society* Cluster 3’s profile is characterized by a vision of society and institutions as trustworthy providers of services and commons (e.g. education, health, security, development). Society is receptive to the demands and needs of people. This vision fosters a generalized feeling of confidence with life, optimism in the future and a sense of agency–what one has to do is to keep oneself within the rules of the game, there being those who take care of handling it for the best. It is worth noting how in the case of this symbolic universe, the trustworthiness attributed to institutions does not mean passivity and dependency. Rather, it works as grounds for a sense of agency: people who identified with this symbolic universe feel able to pursue purposes because they feel part of a system that supports and allows their efforts.
Cluster 4 Moderate pessimism about the future [C4.3], feeling of being immersed in an anomic context [C4.2]; sense of inability to control one’s life [C4.9]; low sense of agency [C4.1], fatalism [C4.10]. People follow utilitarian aims [C4.24]. Agencies are unreliable—in particular those working at the systemic level–public administration [C4.11], healthcare [C4.12], police [C4.20]. On the contrary, school [C4.27] is not considered unreliable, as well as social relationship [C4.2]. Focus on avoiding suffering and survival [C4.15; C4.16; C4.23]. Centrality of belongingness to a powerful [C4.26], familistic [C4.14], amoral [C4.29] network, working as the anchorage of one’s identity [C4.19].	Symbolic Universe 4. *Niche of belongingness* Cluster 4’s profile shares a similar anchorage to the primary network characterizing Cluster 2. Yet, in this case, such an anchorage is combined with a negative generalized connotation of the world outside the primary network–in terms of pessimism in the future, fatalism, untrustworthiness of agencies and institutions. In such a context, the primary network is not a matter of pleasure, an end in itself; rather, it is a necessity responding to the need of finding shelter from and surviving the anomic, threatening outside. Consistently with such a feeling, the primary network is connoted in terms of familistic power (see the agreement with the statements “success depends on forming alliance with stronger people” and “sometimes one has to break the rules to help ones’ loved”). Interestingly, the only institution that is not considered unreliable is the school, namely the only agency among the ones proposed in the questionnaire which is mediated at the level of the local community.
Cluster 5 One’s life goes wrong and will get even worse [C5.2; C5.22]. The same will be true for life in general [C5.4]. The world–and one’s life—belongs to those who have power and use it without scruples [C5.6; C5.26]. No plans and efforts can be made to change things [C5.7; C5.3]. People follow utilitarian aims [C5.33] and one cannot count on them [C5.1]. Social agencies are completely unreliable [C5.11; C5.19; C5.28; C5.35]. The effort to reduce suffering [C5.25] is central along with surviving by following rules [C5.29]—adjusting to living day-by-day [C5.15], affiliating oneself with the winners and powerful people [C5.21].	Symbolic Universe 5. *Others’ world* Cluster 5’s profile outlines a fully negative, even desperate vision of the world–generalized untrustworthiness, sense of impotency, lack of agency, anomie. The world belongs to those who have power–the defeated have only the chance to try to survive day-by-day, surrendering to those with the power to lead the game. Morality and values are a luxury one cannot afford when the only possible concern is to limit the damage.

### Description of the clusters/symbolic universes

**Fig 2:**
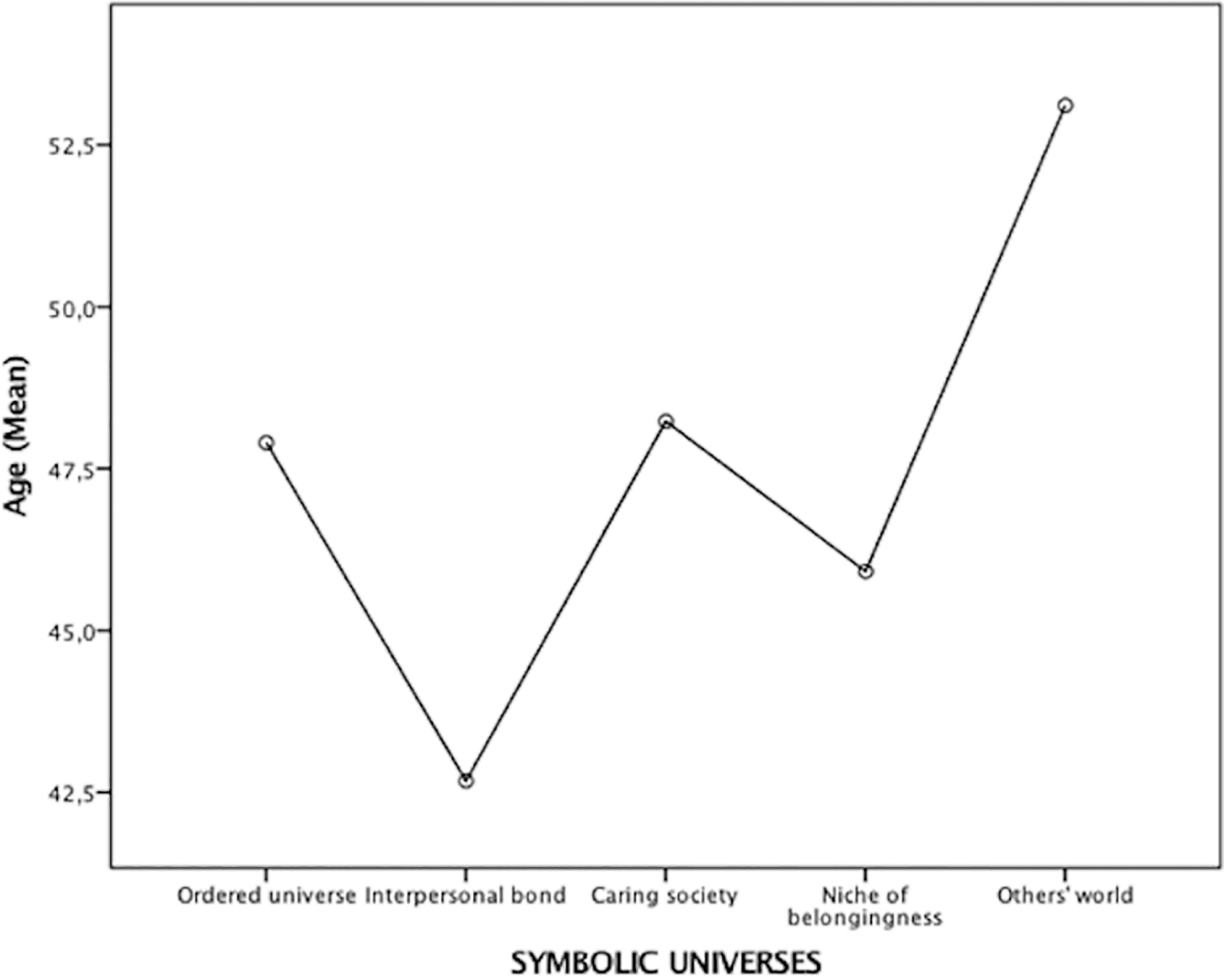
Symbolic universes’ levels of age.

The levels of age of the symbolic universes (i.e., of the clusters of Sample 1 participants; here and henceforth the segments are referred to with the name of the corresponding symbolic universes) were compared by means of ANOVA test. Once subjected to log transformation, age satisfied the condition of homogeneity of variance (Levene test: 1,106; *df* = 4/611; *p* = 0.353). The ANOVA test resulted significant ([*df* = 4/611]: *F* = 3.359; *p* < 0.01). Differences depended on *others’ world*, which is significantly older than *interpersonal bond* and *niche of belongingness* (*p* < 0.01) (cf. Fig 2).

A multinomial logit model was performed in order to assess the dependence structure between the symbolic universes and the set of socio-demographic variables (Sex, Education, Self-assessment of one’s state of health [henceforth: Health], Nucleus [i.e., Size of familiar nucleus], Occupation, Civil Status [Married, Parent, Separated, Widowed, Living with family of origin] and Volunteer activity). The estimated model proved to be statistically significant with a good result of model fitting (chi square = 159,749 *df* = 80; *p* < 0.000). Table 8 reports the overall effects of the model. The variables with an overall statistically significance were two: Education Health (*p* < 0.000).

**Table 8:** Multinomial logit model. Likelihood ratio tests.

*Effect*	*Likehood*	*Chi-square*	*df*	*Sig*
		-1,1	-0,5	-0,6
Intercept	1195.765	0	0	.
Sex	1195.952	0.186	4	0.996
Education	1255.108	59.342	16	0.000
Health	1233.402	37.636	12	0.000
Nucleus	1217.972	22.206	12	0.035
Occupation	1205.545	9.779	12	0.635
Married	1199.771	4.006	4	0.405
Separated	1200.774	5.008	4	0.286
Widowed	1198.073	2.308	4	0.679
Living with family of origin	1199.325	3.56	4	0.469
Parent	1200.967	5.202	4	0.267
Voluntary activity	1199.574	3.809	4	0.432

In order to detect the socio-demographic profiles characterizing the symbolic universes/clusters, we carried out a series of chi-square tests, each of them comparing clusters on one of the socio-demographic characteristic proved to have a significant effect in the multinomial logit model. Symbolic universes/clusters presented significant differences (threshold: *p* < 0.01) on:

- Education (chi square = 64,444 *df* = 16; *p* < 0.01; cf. Table 9): O*rdered universe* (adjusted residual: *AR* = 2) and *interpersonal bond* (*AR* = 3.6) presented a larger proportion of the highest level of Education (>17 years); The opposite occurred with regard to *others’ world* and *niche of belongingness*, which were over-represented in the 6-9-year level (respectively: *AR* = 4.7 and *AR* = 2.5) and under-represented in the highest level (>17 years; *AR* = -3.6 and *AR* = -2.1, respectively). *Caring society* was not marked by differences across levels of education.

**Table 9:** Socio-demographic characteristics of symbolic universes. Education.

	* *	*SYMBOLIC UNIVERSES*					*TOTAL*
		*Ordered universe*	*Interpersonal bond*	*Caring society*	*Niche of belongingness*	*Others' world*	
*< 5 years*	N	1	3	1	7	3	15
	Adjusted Residual	-1.1	-0.5	-0.6	0.9	1.2	
*6–9 years*	N	7	2	7	33	18	67
	Adjusted Residual	-1.5	-4.5	-0.3	2.5	4.7	
*10–13 years*	N	16	22	8	39	15	100
	Adjusted Residual	-0.2	-0.9	-1.2	0.8	1.6	
*14–17 years*	N	26	45	26	57	13	167
	Adjusted Residual	-0.5	0.5	2	-0.5	-1.4	
*> 17 years*	N	41	67	20	58	8	194
	Adjusted Residual	2	3.6	-0.6	-2.1	-3.6	
Total	N	91	139	62	194	57	543

Chi-square = 64.444 *df* = 16; *p* < 0.000

- Health (chi square = 45,535; *df* = 12; *p* < 0.000; Table 10): Differences depended mainly on participants belonging to *others’ world* that tend to describe themselves as having bad (*AR* = 3.9) and very bad (*AR* = 2.8) health conditions.

**Table 10:** Socio-demographic characteristics of symbolic universes. Self-assessment of one’s health condition.

	* *	*SYMBOLIC UNIVERSES*					*TOTAL*
		*Ordered universe*	*Interpersonal bond*	*Caring society*	*Niche of belongingness*	*Others' world*	
*Very Bad—Bad*	N	6	3	2	19	14	44
	Adjusted Residual	-0.6	-2.9	-1.2	1.1	4.7	
*On average*	N	32	56	18	80	18	204
	Adjusted Residual	-0.5	0.9	-1.5	1.3	-1.1	
*Good*	N	33	66	31	65	16	211
	Adjusted Residual	-0.6	2.6	1.9	-1.9	-1.9	
*Very good*	N	21	13	12	32	11	89
	Adjusted Residual	1.9	-2.5	0.6	0	0.5	
Total	N	92	138	63	196	59	548

Chi-square = 45.535; *df* = 12; *p* < 0.000

### Reliability

Table 11 reports the comparisons between the cluster of the main analysis’ response profiles and the corresponding control samples’ response profiles. The comparison was carried out in terms of the percentage of coverage, namely the percentage of items characterizing the cluster of the main analysis that were present in the cluster of the control sample (each cluster of the main analysis was compared with the most similar cluster of the control sample). As one can note, the level of association is variable, however, in most cases quite high. The median level of coverage varies from 63.79% (*Niche of belongingness*) to 77.78% (*Others’ world*).

**Table 11:** Comparison between the response profiles of the clusters of the main analysis and the clusters of the control samples.

*Symbolic universes*	*Sample 1*	*Sample 2*	*Sample 3*	*Sample 4*	*Sample 5*	*Sample 6*	*Sample 7*	*Sample 8*	*Sample 9*	*Sample 10*	*Median*
*Ordered Universe*	76.67	70.00	80.00	76.67	40.00	80.00	76.67	83.33	53.33	63.33	76.67
*Interpersonal bond*	68.00	64.00	56.00	68	80.00	64.00	68	64.00	84.00	72.00	68.00
*Caring* *society*	35.29	76.47	47.06	70.59	85.29	61.76	91.18	67.65	47.06	41.18	64.71
*Niche of belongingness*	44.83	79.31	44.83	65.52	72.41	58.62	79.31	62.07	65.52	44.83	63.79
*Others' world*	85.19	81.48	66.67	77.78	77.78	66.67	74.07	77.78	62.96	85.19	77.78

Each cell holds the percentage of items being common between the *ith* cluster and the more similar *ith* sample's cluster

### Audience segmentation

Table 12 shows the size of the segments of respondents corresponding to the 5 symbolic universes, over the whole Sample 2. Given that Sample 2 is not proportioned relative to the size of the countries’ population (cf. sub-paragraph: Sample 2), both the row and the weighted by population size percentage are reported (the difference between the two results resulted marginal). The largest segment was *niche of belongingness* (33.71%), followed by *interpersonal bond* (23.98%) and *ordered universe* (22.03%); the smallest segments were *caring society* (10.21%) and *others’ world* (10.12%).

**Table 12:** Audience segmentation. Distribution of symbolic universes (Sample 2).

*SYMBOLIC UNIVERSES*	*N over the whole sample*	*% over the whole sample*	*Weighted % over Countries*
*Ordered universe*	303	17.23	22.03
*Interpersonal bond*	418	23.76	23.98
*Caring society*	195	11.09	10.21
*Niche of belongingness*	632	35.93	33.71
*Others' world*	211	12.00	10.12
*Total*	1759		

**Fig 3:**
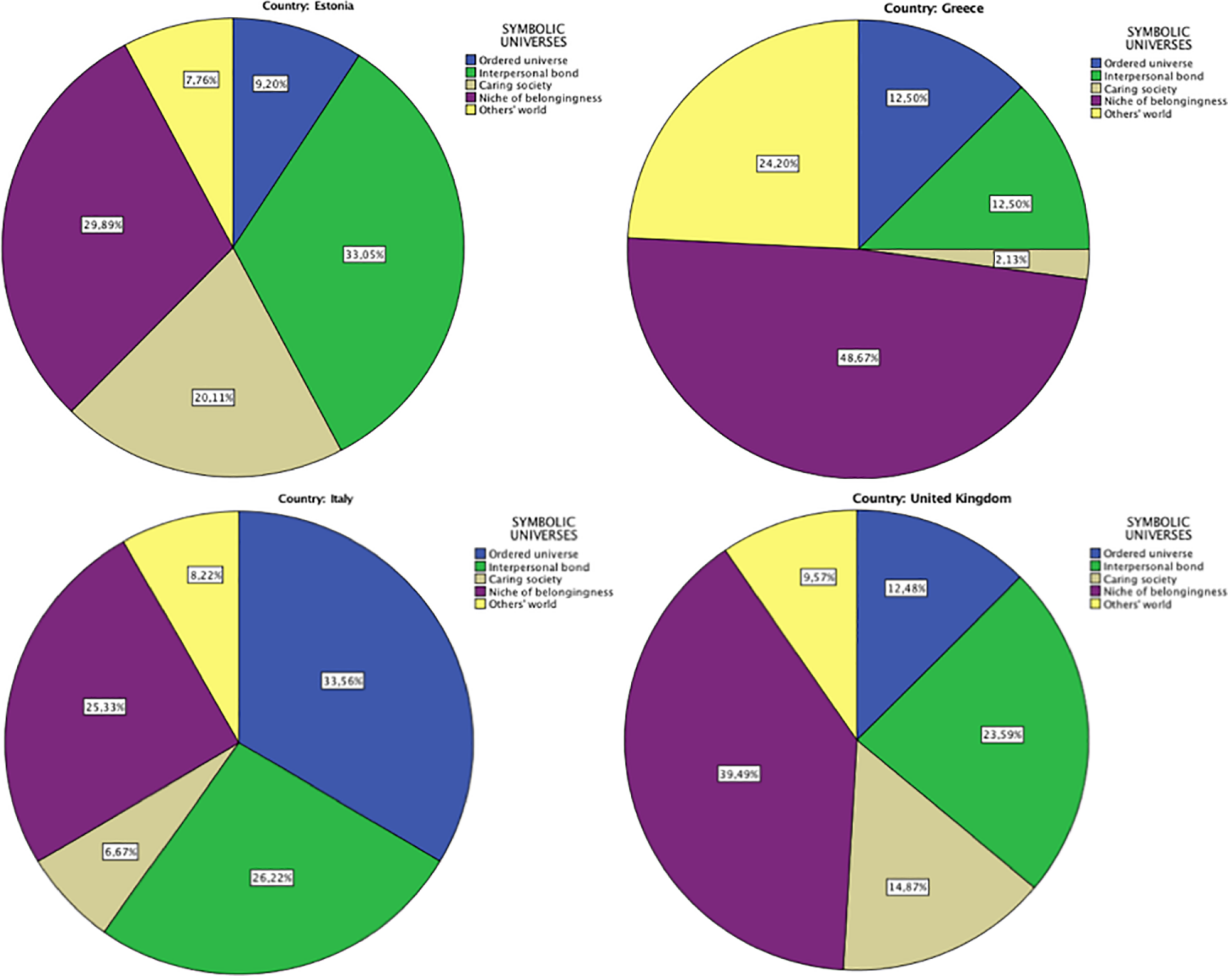
Audience segmentation. Within country distribution of symbolic universes.

Table 13 reports the distribution of the segments within each country (see also Fig 3). The distribution of segments was significantly different between countries (chi-square = 294.128; *df* = 12; *p* < 0.01). As showed by the adjusted residuals,

- Estonia presented a higher proportion of *interpersonal bond* (*AR* = 4.5) and *caring society* (*AR* = 6) and lower proportions of the other symbolic universes.
- Greece presented a higher incidence of *niche of belongingness* (*AR* = 5.8) and *others’ world* (*AR* = 8.2) and lower proportions of the other symbolic universes.
- Italy presented a higher proportion of *ordered universe* (*AR* = 10.6), and a lower incidence of *others’ world* (*AR* = -2.9), *caring society* (*AR* = -3.5) and, above all, *niche of belongingness* (*AR* = -5.4).
- The United Kingdom presented a higher proportion of *caring society* (*AR* = 3.6) and *niche of belongingness* (ar = 2.2) and a lower proportion of *others’ world* (*AR* = -2.2) and *ordered universe* (*AR* = 3.7).

**Table 13:** Distribution of symbolic universes within countries.

		*COUNTRY*				
SYMBOLIC UNIVERSES		*Estonia*	*Greece*	*Italy*	*UK*	TOTAL
*Ordered universe*	N	32	47	151	73	303
	Adjusted Residual	-4.4	-2.7	10.6	-3.7	
*Interpersonal bond*	N	115	47	118	138	418
	Adjusted Residual	4.5	-5.8	1.4	-0.1	
*Caring society*	N	70	8	30	87	195
	Adjusted Residual	6	-6.2	-3.5	3.6	
*Niche of belongingness*	N	104	183	114	231	632
	Adjusted Residual	-2.6	5.8	-5.4	2.2	
*Others' world*	N	27	91	37	56	211
	Adjusted Residual	-2.7	8.2	-2.9	-2.2	
	Total	348	376	450	585	1759

## Discussion

### Content and characteristics of the symbolic universes

Each cluster’s response profile demonstrated a consistent pattern of meanings spreading over different domains of experience proposed by the VOC questionnaire. Each pattern is composed of affectively homogeneous meanings, regardless of the semantic linkage between the objects subjected to interpretation (e.g. the future, the reliability of agencies, politicians, immigrants and so forth). This is consistent with the theoretical interpretation of these patterns as markers of symbolic universes, the latter intended as affect-laden, generalized meanings.

With regards to the characterization of people associated with symbolic universes, one can observe how they are consistent with the way the latter have been interpreted: symbolic universes representing a negative approach to reality (*others’ world*, *niche of belongingness*) are associated with more critical socio-demographic characteristics -older age, lower levels of education, worse self-evaluation of health conditions (the latter characteristics with *others’ world* only).

Finally, the 5 partitions obtained by the CA resulted sufficiently reliable with regards to their independence from sampling. Incidentally, this is consistent with SCPT view of them as systems of generalized, affect-laden meanings substantiating very basic, embodied worldviews rooted in the cultural milieu.

### Symbolic universes as forms of semiotic capital

Symbolic universes lend themselves to be viewed in terms of semiotic capital. With semiotic capital we mean repertoires of generalized meanings that work as resources for civil and socio-economic development. Here we highlight the affective laden, pre-reflective, generalized and embodied valence of the generalized meanings semiotic capital consists of. We prefer the term “semiotic capital” to the more used *symbolic capital*, because the latter is more strictly related to the Bourdieu’s theory and intended in terms of prestige and celebrity -“degree of accumulated prestige, celebrity or honour and is founded on a dialectic of knowledge and recognition” ([38], p. 7). Rossolatos [39] has recently used the term “semiotic capital” in a similar way adopted here.

Needless to say, semiotic capital is related to social capital [40]; see also [41–43], which occurs in societies in two different main forms: bonding social capital includes networking between homogeneous groups, it is characterized by shared norms and collaboration, and provides protection and safety to both individuals and groups. Bridging social capital includes networking between heterogeneous and diverse groups and it is based on the exchange of information, ideas, and resources. A third form -linking social capital- has also been proposed [44], to refer to ties and relationships that connect different levels of the social hierarchy.

However, semiotic capital requires distinction from social capital too. Indeed, the embodied, generalized meanings it consists of lead to consider symbolic universes as the cultural, affective source of a sense of trust, the quality of institutions, and networking (i.e. the most referred forms of social capital). This view is not inconsistent with a non-functionalist interpretation of social capital. For instance, Ostroom and Ahn [1] state that:

“Trust cannot always be explained entirely by the incentives embedded in the structure of social interactions (…) We emphasize that individuals’ intrinsic values are an independent reason for behaving cooperatively and reserve the term trustworthiness primarily to refer to such non-selfish motives” (pp. 25–26)

Symbolic universes represent a way for modelling “intrinsic values” as the expression of the position of the individual within a cultural milieu. Rather than assuming “intrinsic values” as a primitive datum, the latter can be understood as circularly connected to the social processes they themselves help bring about.

According to the perspective outlined above, due to their content and socio-demographic profile, two symbolic universes -*ordered universe*, *caring society -*can be viewed as functional forms of *semiotic capital*. Indeed, both of them are characterized by reference to a super-order, systemic dimension of social life that enables people to recognize and give relevance to the relation between the individual sphere of experience and the sphere of collective life that goes beyond the experience of oneself and the primary bond (i.e., family relatives, close friends). In one case (*ordered universe*), such a reference consists of the anchorage to an axiological belief about how the world works and therefore how things cannot but proceed the way they are; in the other case (*caring society*) the system is represented in terms of institutions and agencies working as providers of commodities (resources and services), whose consumption feeds the individual’s autonomy. Accordingly, the universalistic breadth of ordered universe leads to see it as a worldview feeding what is known as *bridging* forms of social capital, whereas the functional anchorage to the structural dimension of social life (institutions, agencies) characterizing caring society leads to an association of this symbolic universe to *linking* forms of social capital–namely social capital consisting of hierarchical, top-down relationships (with regards to the notion of linking social capital, see [44]).

Regarding *interpersonal bond* and *niche of belongingness*, these can be seen as a source of what is known as *bonding* social capital–i.e. what feeds the in-group identity and cohesion. On the one hand, *niche of belongingness* can be seen as a critical form of bonding capital -a worldview leading to put bridging and bonding forms of social life in conflict with each other: the *us* meant as a protection from *them*. One can add that such an opposition seems constitutive: us consists of what is threatened by what comes from the outside. On the other hand, the positive connotation of the world expressed by *interpersonal bond* seems to be reached in terms of a sort of affective hedonism, namely in terms of the absolutization of the emotional networking and the backgrounding of any reference to what is beyond it.

Finally, the analysis has shown that *others’ world* is a sort of *semiotic black hole*: it leads to experience being lived in absolutely negative terms–for those who are characterized by this symbolic universe, the world appears full of extraneous and aggressive events, a jungle. From within this worldview no positive elements and no resource can be seen. Any critical aspect is felt as a further sign of the totally negative reality. In the dark night everything cannot but be dark -there is no room for variability, modulation, or time, no possibility for changing what is inherently and fully alien. All that remains is the reactive acceptance of existence as a way of surviving.

### Audience segmentation

Taking the sample as a whole, it is clearly critical that the two symbolic universes we have interpreted as *semiotic capital* represent about 1/3 of the country-samples in the analysis. The cultural picture that emerges is a society divided in three sections: 1/3 (*ordered universe* and *caring society*) able to make sense of the world in terms of universalistic rules and values as well as trust and agency; 1/3 closed within a defensive identification with their identity group; and 1/3 entangled and/or entrapped in the present, which is idealized (*interpersonal bond*) or, conversely, regarded as the worst of possible worlds (*others’ world*).

If one considers the tripartite distinction detailed above, segments’ distribution among countries result rather similar (with the exclusion of the case of Greece). Indeed, in Italy, UK and Estonia the three sections–i.e. the systemic worldviews (*ordered universe* and *caring society)*, the defensive identity-focused worldview (*niche of belongingness*), and the symbolic universes leading to identification with emotional life (i.e. *interpersonal bond* and *others’ world*) tend to distribute in somehow similar ways within each country–Italy: 30-30-40; Estonia: 40-25-35; UK: 27-39-33.

The case of Greece was quite different. The systemic worldviews in Greece correspond to about 14% of the sample (within it, *caring society* 2%), almost half of the sample is covered by the *niche of belongingness* and the third section (the area of the emotional reaction) is comprised of the anomic symbolic universe (*others’ world*) that alone represents 1/4 of the population. The fact that the European country that underwent the most violent socio-economic crisis is characterized by an incidence of critical -defensive, anomic, reactive -symbolic universes that is far higher than that shown by the other three countries provides food for thought (for a discussion on the circular relation between contextual conditions and cultural dynamics, see [10]).

### Trends and future questions regarding symbolic universes

The analysis on the symbolic universes and the audience segmentation discussed above raise several issues concerning the relation between the cultural milieu and socio-political dynamics that have spanned Europe over the last decade. In this and the following subparagraphs we propose some speculative considerations regarding these issues. In so doing, our purpose is to outline the agenda of a more general research program aiming at understanding the role played by cultural dynamics in the European socio-political and institutional crisis and its further development.

Firstly, it is worth asking: *Is the size of the segments estimated in the present study a changing structure*, *or has it been stable over the last decade?*

Our results do not provide a direct answer to this question. Thus, we limit ourselves to propose a tentative hypothesis that requires further systematic inquiry. In absence of historical data that could be directly comparable with the current audience segmentation, we refer to the 2004 European Value Survey’s item concerning the level of satisfaction with one’s own life (cf. www.europeanvaluesstudy.eu*)*. We do so because the level of dissatisfaction with one’s own life is a constitutive component of the two symbolic universes consisting of a critical worldview–*niche of belongingness* and *others’ world*–discriminating them from the others (indeed, *ordered universe*, *interpersonal bond* and *caring society* are characterized by satisfaction with own life). Thus, the proportion of people being disaffected with own life lends itself as an indirect indicator of the size of the segments *niche of belongingness* and *others’ world*.

Once measured as proposed, two main elements emerge.

Firstly, the size of the two critical segments increased from 2004 to 2016, even if with relevant variation in the 4 countries involved in the current analysis. Indeed, comparing with the 2016 audience segmentation, the estimation of the 2004 size results as follows:

- the same in Estonia (2004 percentage of dissatisfied people: 37%; 2016 aggregate percentage of *niche of belongingness* and *others’ world*: 37%);
- is rather lower in Italy (2004: 34%; 2016: 40%),
- is quite lower in UK (2004: 28%; 2016: 48%) and even more in Greece (2004: 34%; 2016: 73%).

Second, there are reasons to conjecture that in Italy and above all in UK the increase of the two critical segments view of the world is due to the growth of the defensive, identity focused segment–i.e. *niche of belongingness*. Indeed, the other critical segment (*others’ world*) shows a rather low proportion in both countries and, above all, such proportion is similar to that of Estonia, that is, the country where the global level of critical segments remained stable between 2004 and 2016. The Greek dynamics seem to be different: in this case the dramatic increase of the two critical segments from 2004 to 2016 seems due to both segments, as signalled by the fact that in this country the 2016 size of both *niche of belongingness* and *others’ world* are far larger than that of the other three countries (whereas in the 2004 the Greek level of dissatisfaction was similar to that of Italy and Estonia).

### Scenario transformations and cultural milieu

The reconstruction of the dynamics of symbolic universes, though tentative, raises a second question: *How are such dynamics related with the social*, *economic*, *political and institutional processes marking recent European history?*

It is plausible that the current state of the cultural milieu has been affected by what has been happening in Europe and in the world over the last decades. The high incidence of critical segments in Greece provides support to this view. On the other hand, this view is consistent with the diffusion of a dramatic generalized worsening of perceived quality of life over last years. Yet, the indirect retrospective reconstruction of the segments’ size leaves room for a less obvious interpretation: as showed by 3 (Estonia, Italy, UK) out of 4 countries’ segmentation: the cultural milieu does not seem to respond mechanically to the crisis. As observed above, in Italy the two critical segments seem to have increased between 2004 and 2016, yet less than in UK, despite the fact that the former country was effected by the socio-economic crisis in a more intense and durable way than the latter. Thus, one is allowed to consider the relation between culture and society in a more complex way: social dynamics affect the cultural milieu, yet this happens in a way that depends not only on the content and intensity of the social dynamics, but on the form and diffusion of the semiotic capital within the country’s cultural milieu too. According to this interpretative standpoint, one is led to speculate that people made sense of the critical scenario conditions through the mediation of the semiotic resources available within their cultural milieu: in Italy mainly through the semiotic resources provided by *ordered universe*, in Estonia by *caring society*, in the UK and above all in Greece by *niche of belongingness*.

### Symbolic universes and the current socio-political scenario

SCPT views the relation between cultural milieu and social dynamics as recursive: symbolic universes are both the effect and the determinants of the socio-economic and political-institutional context (for a similar approach, see for instance Uslaner, [45] p. 139; the author uses it for modelling the relation between social trust, economic inequality, corruption, and quality of the governance). Accordingly, one could ask: *What role do symbolic universes play in fostering and/or constraining current and future socio-economic and institutional scenarios?*

The audience segmentation represents an interpretative framework for addressing such a question. Indeed, it shows how the negative, anomic experience of the world induced by the socio-political crisis tends to be addressed and assimilated in terms of *niche of belongingness*, whose symbolic specificity consists of defensive identification with the in-group, intended as a barrier against the persecutory outside. This process seems to happen less in Estonia and partially in Italy, countries where the antagonizing effect of forms of functional (linking and/or bridging) semiotic capital seem to work.

The polarization of the in/outgroup conflict as an affective, defensive mechanism used to cope with an uncertain and perturbing context has been widely studied in psychoanalysis [46] as well as in philosophy [47], social psychology (starting from [48]) and sociology (e.g. [49, 50]). The emotional construction of the context in terms of a persecutory entity allows one to transform the *absence* (i.e., the lack of control and capacity of making sense of the hyper-complex environment) to a *presence* (i.e., the persecutory other that is the cause of the lack) that can be represented and therefore addressed somehow, at least at the psychological level. In so doing, the actor is able to restore one’s own sense of agency and stability, though at the cost of an emotional simplification of reality.

The hypothesis of the *niche of belongingness* as the most available semiotic defence from the anomic disintegration of society requires more systematic validation. This being said, one cannot but observe how it seems to work as a consistent interpretative framework for the several socio-institutional phenomena of intergroup conflict’s radicalization referred to in the introduction. Due to the introductive valence of this discussion, in what follows we outline some general ideas that follow from this interpretative perspective.

Firstly, once framed in terms of salience of *niche of belongingness*, the forms of radicalization of intergroup conflict–regardless of the social and ethical valence of their content (i.e. xenophobia and religious crime do not have the same valence of identification with the local community)–acquire the value of acts of meaning and searching for identity cohesion. These are aimed at fulfilling the basic need of making the experience of a more and more chaotic, world representable and understandable. Accordingly, we propose a particular focus on the UK, the country whose people decided to quit Europe, where it is *niche of belongingness* and not *others’ world* that is the largest segment. In other words, these forms have to be recognized as semiotic solutions to the crisis. One can discuss if such solutions are worse than the problem they address, however, from the subjective perspective of the sense-maker, these are a way of restoring a sense of meaningfulness. Accordingly, it is hard to think that any institutional-political offering could appeal only to its inherent functional quality. Rather, we contend that its appeal is also and mainly a matter of its capacity to provide an alternative solution to the demand of sense raised by the experience of the anomic, ungraspable context.

Secondly, the relevance of identity motivation is recognized in the main psychosocial models that explain electoral behaviour through partisanship [51]. The reference to *niche of belongingness* does not contradict this view. Rather, it leads to a recognition of how in the current processes of radicalization of the intergroup conflict, the threatening, persecutory, connotation of the other is constitutive–the us finds definition in its being in conflict with who is other-than. Thus, for instance, a large segment of the population vote in accordance with their identity motivation. Yet, what is peculiar of contemporary political electoral choice is that identity motivation is less and less positive–i.e. pro someone/something, aimed at affirming a system of values associated with a certain social group–and more and more oppositional and in negative–i.e. against someone/something, e.g., for getting rid of the political caste (for a recent analysis of this kind see Cramer [52]; see also the concept of "negative politics" in Rosanvallon [53]’s theory of counter-democracy).

## Conclusions

This paper reported the framework, method and main findings of an analysis of cultural milieus in (a set of) European societies as well as their interpretation in light of the current socio-institutional European situation. The main findings can be summarized as follows.

Firstly, we identified 5 symbolic universes, each of them consisting of a basic, embodied, affect-laden, generalized worldviews. Four of these -*ordered universe*, *interpersonal bond*, *caring society*, *niche of belongingness-* can be interpreted as reflecting the salience of a specific anchorage–the ethical, axiomatic framework; the interpersonal bond; the institutions and structures of the social system; the system of belongingness, respectively. The other symbolic universe, *others’ world*, can be seen as fostered by the failure, or absence, of these anchorages. In consequence, this experience of the world acquires the form of a generalized anomic reaction that sees everything in a negative, fatalistic way.

Secondly, we have proposed that *ordered universe* and *caring society* be considered as two forms of semiotic capital, namely generalized meanings grounding people’s capacity to recognize the “rules of the game” (what we have called: the systemic level of social life) and therefore to foster social and civic development.

Thirdly, we have analysed the socio-demographic profiles characterizing each symbolic universe. This characterization was consistent with the interpretation of the symbolic universes, that is, symbolic universes interpreted as critical resulted associated with more negative socio-demographic characters (e.g. low level of education).

Fourthly, the symbolic universes were used for segmenting the four country samples. Their distribution was variable between countries. However, with the exception of the Greek sample, the country samples resulted divided in three macro-segments that were remarkably similar: 1/3 were characterized in terms of semiotic capital, 1/3 identified defensively with the in-group as a protection against the external world (*niche of belongingness*), and 1/3 characterized in terms of here and now emotional experiences consisting of an idealization of the relational life (*interpersonal bond*) or the surrender worst possible world (*others’ world*).

Finally, considerations have been provided at a more speculative level regarding the retrospective reconstruction of the incidence of symbolic universes as well as the interplay between them and past, present and future socio-institutional scenarios.

### Limitations and further direction of the research

Some main limitations of the study ought to be highlighted.

First, the survey adopted a convenience sample as source of data (with the exception of the UK). Needless to say, the combination of the use of an on-line procedure and the adoption of a convenience sample exposes the survey to significant limitations. Indeed, the composition of the population of respondents is affected by accessibility to the internet and level of commitment. Consequently, the convenience sample does not allow control for representativeness of the samples. The post-hoc procedures of randomization adopted, together with the post-hoc analysis of reliability were designed to improve the quality of results, by increasing of the balance of the sample. However, further analyses are needed to estimate the level of ecological validity of results and to control for it in a fully efficacious way. On the other hand, according to the theoretical framework of the study–more particularly the generalized valence of the meanings comprising the symbolic universes [10, 18, 27]—one can expect that the fact of using a non-randomized sample should not have affected the ecological validity of the findings.

Second, whereas the audience segmentation was performed on country-subsamples, the identification of the symbolic universes focused on the sample comprised of respondents belonging to several European countries taken as a whole. Needless to say, this choice assumes European societies as a cultural entity, having a consistent inner organization. Yet this is an assumption devoid of empirical evidence. The findings could therefore be a methodological artefact–namely the map of cultural milieus of European societies could be the consequence of the fact of considering it as an organic whole, rather than the reflection of a state of fact. Only analyses focused on single countries can provide support to the methodological choice of considering European societies as a sufficiently homogeneous cultural milieu that can be studied as a whole. In fact, we have collected such findings and these are consistent with our choice; however, also in this case, further analysis is required.

Finally, the findings of our study point to the requirement for studying further how the symbolic universes, on the one hand, are associated with psychological and socio-economic conditions, and, on the other hand, how they shape concrete, situated attitudes and behaviour in the various domains of social life (e.g. attitudes towards public policies and electoral choice). This level of analysis is strategic both for testing the construct validity of the map of the cultural milieu (i.e. the SCPT basic assumption that the cultural dimensions play a main role in social life) and for highlighting how the knowledge of the cultural milieu could be strategic for policy makers engaged with the design and implementation of policies.

## Supporting information

S1 Text Questionnaire Views of Context (VOC). (PDF)

S1 Table Multiple correspondence analysis. Description of the 3 main factorial dimensions.
(DOCX)

S1 Fig Position of the symbolic universes on the MCA main factorial dimensions. Factor 1 vs. Factor 2.
(PDF)

S2 Fig Position of the symbolic universes on the MCA main factorial dimensions. Factor 1 vs. Factor 3.
(PDF)

## References

[1] Ostrom E, Ahn T K. The meaning of social capital and its link to collective action In Swendsen G T Swendsen G L H, editors. Handbook of Social Capital. The Troika of Sociology, Political Science and Economics. Cheltenham, UK: Edward Elgar; 2009 pp. 17–31.

[2] Mitchell S A. Relational Concepts in Psychoanalysis. An Integration. Cambridge Mass: Harvard University Press; 1988.

[3] Salvatore S, Freda M F. Affect, Unconscious and Sensemaking. A Psychodynamic, Semiotic and Dialogic Model. New Ideas in Psychol. 2011; 29: 119–135.

[4] Salvatore S, Zittoun T. Outlines of a psychoanalytically informed cultural psychology In Salvatore S, Zittoun T, editors. Cultural Psychology and Psychoanalysis in Dialogue. Issues for Constructive Theoretical and Methodological Synergies. Charlotte, NC: Information Age; 2012 pp. 3–46.

[5] Laura-Grotto R P, Salvatore S, Gennaro A, Gelo O. The unbearable dynamicity of psychological processes: Highlights of the psychodynamic theories In Valsiner J, Molenaar P, Lyra M, Chaudhary N, editors. Dynamics process methodology in the social and developmental sciences. New York: Springer; 2009 pp. 1–30.

[6] Salvatore S, Lauro-Grotto R, Gennaro A, & Gelo O. Attempts to grasp the dynamicity of intersubjectivity In Valsiner J, Molenaar P, Lyra M, Chaudhary N, editors. Dynamics process methodology in the social and developmental sciences. New York: Springer; 2009 pp. 171–190.

[7] Salvatore S, Tschacher W. Time dependency of psychotherapeutic exchanges: the contribution of the theory of dynamic systems in analyzing process. Frontiers in Psychology. 2012; 3:253 doi: 10.3389/fpsyg.2012.00253 22848205 10.3389/fpsyg.2012.00253 PMC3404413

[8] Thelen E, Smith L. A dynamic systems approach to the development of cognition and action. Cambridge: MIT Press; 1994.

[9] Rosa A. Act of Psyche In Valsiner J, Rosa A, editors. The Cambridge Handbook of Sociocultural Psychology Cambridge: Cambridge University Press; 2007 pp. 205–237.

[10] Salvatore S. Psychology in black and white The project of a theory-driven science. Charlotte NC: Information Age Publishing; 2016.

[11] Valsiner J. Culture in Minds and Societies Foundations of Cultural Psychology. New Delhi: Sage Publications; 2007.

[12] Valsiner J. An Invitation to Cultural Psychology. London: Sage Publications; 2014.

[13] Valsiner J, Rosa A, editors. The Cambridge Handbook of Sociocultural Psychology. Cambridge: Cambridge University Press; 2007.

[14] Salvatore S. The cultural psychology of desire In Valsiner J, Marsico G, Chaudhary N, Sato T, Dazzani, editors. Psychology as the Science of Human Being. The Yokohama Manifesto. Annales of Theoretical Psychology. Heidelberg New York Dordrecht London: Springer; 2016 pp. 33–49.

[15] Berger P L, Luckmann T The Social Construction of Reality: A Treatise in the Sociology of Knowledge. Garden City, NY: Anchor Books, 1966.

[16] Cobern W W, Aikenhead G. Cultural Aspects of Learning Science. Scientific Literacy and Cultural Studies Project (13); 1997 Available from: http://scholarworks.wmich.edu/science_slcsp/13.

[17] Venuleo C, Mossi P G, Salvatore S. Educational subcultures and dropping out in higher education. A longitudinal case study. Studies in Higher Education; 2016: 41(2), 321–342, doi: 10.1080/03075079.2014.927847

[18] Salvatore S, Venuleo C. Field dependency and contingency in the modelling of sensemaking. Papers on Social Representation [On Line Journal]. 2013 22(2); 21.1–21.41.

[19] Salvatore S, Tonti M, Gennaro A. How to model sensemaking. A contribution for the development of a methodological framework for the analysis of meaning In Han M, Cunha C, editors. The Subjectified and Subjectifying Mind. Charlotte, NC: Information Age Publishing, 2017 pp. 245–258.

[20] Tonti M, Salvatore S. The Homogenization of Classification Functions Measurement (HOCFUN): A method for measuring the salience of emotional arousal in thinking. Am J Psychol. 2015; 128(4): 469–483. 26721175 10.5406/amerjpsyc.128.4.0469

[21] Roser-Renouf C, Maibach E, Leiserowitz A, Feinberg G, Rosenthal S, Kreslake J. Global Warming's Six Americas, October, 2014: Perception of the Health Consequences of Global Warming and Update on Key Beliefs Yale University and George Mason University New Haven, CT: Yale Project on Climate Change Communication; 2014 Available from http://climatecommunication.yale.edu/wp-content/uploads/2015/03/Six-Americas-October-2014.pdf

[22] Jegede O J, Aikenhead G S. Transcending cultural borders: implications for science teaching [Electronic version]. Journal for Science & Technology Education. 1999; 17(1), 45–6.

[23] Triandis H C. The psychological measurement of cultural syndromes. Am Psychol. 1996; 51: 407–415.

[24] Campo S, Askelson N M, Carter K D, Losch M. Segmenting Audiences And Tailoring Messages: Using The Extended Parallel Process Model And Cluster Analysis To Improve Health Campaigns. Soc Mark Q. 2012; 18(2): 98–111. doi: 10.1177/1524500412450490

[25] Carli R, Salvatore S. [The image of psychology: A study on the Lazio's population]: L’immagine della psicologia: Una ricerca sulla popolazione del Lazio. Roma: Kappa; 2001.

[26] Venuleo C, Salvatore S, Mossi P G. The role of cultural factors in pathological gambling. J of Gambl Stud. 2015; 31(4), 1353–76. doi: 10.1007/s10899-014-9476-z 24970696 10.1007/s10899-014-9476-z

[27] Ciavolino E., Redd R., Avdi E., Falcone F., Fini V., Kadianaki I., et al Views of Context. An instrument for the analysis of the cultural milieu. A first validation study. Electronic Journal of Applied Statistical Analysis, 10 (2), 599–628

[28] Guidi M, Salvatore S. Parents’ images of their Children’s School System In Iannaccone A, Komatsu K, & P Marsico P, editors. Crossing Boundaries. Intercontextual dynamics between family and school. Charlotte, NC: Information Age Publication; 2013 pp. 271–300.

[29] Carli R, Paniccia R M. [Psychology of training]: Psicologia della formazione. Bologna: Il Mulino; 1999.

[30] Venezia, A. [Medicine in contemporary society. A semiotic culturalist analysis]: La medicina nella società contemporanea. Un’analisi semiotico culturalista Dissertation Thesis. University of Salento (Italy); 2016.

[31] Mannarini T, Nitti M, Ciavolino E, Salvatore S. The role of affects in culture-based interventions: Implications for practice. Psychology. 2012; 3: 569–577. doi: 10.4236/psych. 2012.38085

[32] Carli R. Pagano P. [Saint Lawrence. The culture of the neighborhood and the relationship with psychology] San Lorenzo La cultura del quartiere e il rapporto con la psicologia. Roma: Edizioni Kappa; 2008.

[33] Fini V, Fregonese C, Guidi M. [Planning the development: of whom? How? Why?]. Pianificare lo sviluppo: di chi? Come? Perché? Rivista Scritti di Gruppo. 2011; 2, Available from http://www.associazioneppg.it/co/ottobre_2011.pdf.

[34] Carli R, Paniccia R M. [Psychosociology of the Traffic: the roman case] Psicosociologia del traffico: il caso romano. Roma: Capitolio; 1999.

[35] Carli R, Paniccia R M, Atzori E, Cinalli S, Brescia F, Mazzeo G, et al [The psychosocial risk in a Roman hospital: the relationship between Local Culture and satisfaction in the Hospital Complex Company San Filippo Neri]: Il rischio psicosociale in un Ospedale romano: il rapporto tra Cultura Locale e soddisfazione nell’Azienda Complesso Ospedaliero San Filippo Neri. Rivista di Psicologia Clinica. 2012; 1: 76–90. Available from http://www.rivistadipsicologiaclinica.it/ojs/index.php/rpc/article/view/134.

[36] Osgood C E, Suci G J, Tannenbaum P H. The measurement of meaning. Urbana: University of Illinois Press; 1957.

[37] Abdi H., & Valentin D. (2007). Multiple correspondence analysis In Salkind N.J. (Ed.): *Encyclopedia of Measurement and Statistics*. Thousand Oaks (CA): Sage pp. 651–657.

[38] Thompson J. B. Introduction In Bourdieu P. Language and Symbolic Power. [Edited and Introduced by Thompson John B.] Cambridge: Harvard University Press; 1991.

[39] Rossolatos G. Before the consummation what? On the role of the semiotic economy of seduction. J Media Cult Stud. 2016; 30(4): 451–465.

[40] Driessens O. Celebrity capital: Redefining celebrity using field theory. Theory Soc. 2013; 42 (5): 543–560.

[41] Coleman J S. Foundations of Social Theory. Cambridge, MA: Harvard University Press; 1990.

[42] Putnam R D. Making Democracy Work: Civic Traditions in Modern Italy. Princeton, NJ: Princeton University Press; 1993.

[43] Swendsen G T, Swendsen G L H, editors. Handbook of Social Capital. The Troika of Sociology, Political Science and Economics. Cheltenham, UK: Edward Elgar; 2009.

[44] Woolcock M. The place of social capital in understanding social and economic outcomes, ISUMA Canadian Journal of Policy Research. 2001; 2(1): 11–17.

[45] Uslaner E. M. Corruption In Swendsen G T Swendsen G L H, editors. Handbook of Social Capital. The Troika of Sociology, Political Science and Economics. Cheltenham, UK: Edward Elgar; 2009 pp. 127–141.

[46] Klein M. Contribution to Psychoanalysis, 1921–1945. New York: Mac Graw-Hill; 1967.

[47] Pulcini E. Care of the world. Fear, responsibility and justice in the global age. Dordrecht, NL: Springer; 2012 [or. ed. 2009].

[48] Tajfel H, Turner J C. An integrative theory of intergroup conflict In Austin W G, Worchel S, editors. The social psychology of intergroup relations. Monterey, CA: Brooks Cole; 1979 pp. 33–

[49] Bauman Z. Community. Seeking safety in an insecure world. Cambridge: Polity Press; 2001.

[50] Castells M. The power of identity. Malden-Oxford, UK: Blackwell; 1997.

[51] Green D, Palmquist B, Schickler E. Partisan hearts & minds: Political parties and the social identities of voters. New Haven: Yale University Press; 2002.

[52] Cramer K. The Politics of Resentment: Rural Consciousness in Wisconsin and the Rise of Scott Walker. ILL: University of Chicago Press; 2016.

[53] Rosanvallon P. Counter-democcary. Politics in the age of distrust. Cambridge, UK: Cambridge University Press; 2008 [or. ed. 2006].

