# Correction: Symbolic universes between present and future of Europe. First results of the map of European societies' cultural milieu

**DOI:** 10.1371/journal.pone.0200223

**Published:** 2018-06-28

**Authors:** Sergio Salvatore, Viviana Fini, Terri Mannarini, Giuseppe Alessandro Veltri, Evrinomi Avdi, Fiorella Battaglia, Jorge Castro-Tejerina, Enrico Ciavolino, Marco Cremaschi, Irini Kadianaki, Nikita A. Kharlamov, Anna Krasteva, Katrin Kullasepp, Anastassios Matsopoulos, Claudia Meschiari, Piergiorgio Mossi, Polivios Psinas, Rozlyn Redd, Alessia Rochira, Alfonso Santarpia, Gordon Sammut, Jaan Valsiner, Antonella Valmorbida, on behalf of the Re.Cri.Re. Consortium

There is an error in affiliation 12 for authors Anastassios Matsopoulos and Polivios Psinas. Affiliation 12 should be: School of Education, Department of Preschool, University of Crete, Rethimno, Crete, Greece.
