# Supplementary material for: Symbolic universes between present and future of Europe. First results of the map of European societies' cultural milieu
Source: PLoS One. 2018 Jan 3;13(1):e0189885. doi: 10.1371/journal.pone.0189885 (PMC5752019; doi:10.1371/journal.pone.0189885)
Supplement: S1 Text — (PDF) [file pone.0189885.s001.pdf]

S1 Text. Questionnaire “Views of Context” (VOC)

RE.CRI.RE

BETWEEN THE REPRESENTATION
OF THE CRISIS AND THE CRISIS
OF REPRESENTATION

QUESTIONNAIRE "VOC"
"Views Of Context"

DATE: 10.10.2015
VERSION: 1.7

"This project has received funding from the *European Union's Horizon 2020 research and innovation programme* under grant agreement No 649436".

RE.CRI.RE

VOC - Views Of Context

This questionnaire investigates the ways that people represent the Place where they live and how these representations are associated with their way of thinking and feeling.

The questionnaire is part of a international study aimed at better understanding people's needs, sensibilities and attitudes in order to inform the design of programs and policies more consistent with the cultural specificity of territories.

The results of this study are expected to enable more efficient, efficacious, and culturally sensible participant-centered programs in several fields (e.g. education, health promotion, social cohesion, mobility, safety, labor market).

In order to complete this ambitious goal, your collaborations is precious and necessary.

Your participation will help us to include in the survey the local territory where you live. In doing so, the results of the study will also concern the place where you live and this might increase the representativeness and validity of programs and policies informed by our study.

How to complete the questionnaire

The questionnaire will take approximately 30-35 minutes to complete.

There are no right or wrong answers; rather, several options that can give an account of your point of view on the aspects presented.
When answering, you will notice that every word, every sentence, even the simplest, can be understood and interpreted in various ways. Do not worry about that and just **give the first answer that comes to mind.**
When answering the questionnaire, it is best to try to **proceed quickly.**
In most cases, in order to answer you just have to select the box that best corresponds to your point of view.
In some cases, there may be no alternative that fits your point of view exactly. In such cases, we invite you to "force" yourself, and **give your answer anyway**.
The questionnaire is anonymous. **Your responses will not be made public; they will be taken into account together with those of all the other respondents.**

When you have finished the questionnaire, if you want you can leave your e-mail address, so that we can send you the report of the study, once it is produced.

If you have questions about the questionnaire, you can contact:
e-mail: info@recrire.eu

By clicking on the button "Next" you confirm that you have read the information above, that you are over 18 years of age and voluntarily agree to participate in the survey.

RE.CRI.RE

Before you start, we ask for some information.

0.Age (in years)______

00.Sex: Female □
Male □

000.The place where you live mainly

Country ________________________
State/Region ________________________
City/Town ________________________
District/Neighbourhood ________________________

Session 1 – THE PLACE WHERE YOU LIVE

CONSIDER THE PLACE WHERE YOU LIVE (YOUR CITY, TOWN, VILLAGE OR NEIGHBORHOOD AS YOU PREFER)

You will find listed below some Agencies and Services present in it. Please indicate how reliable each of them is, in your opinion.

	Not at all reliable	Not very reliable	Quite reliable	Very reliable
Public transport				
Health care services				
Police				
Schools				
Public Administration				
Companies				

Below you will find some statements that refer to the Place where you live, intended as a community of people residing in the same territory. We ask you to respond to each of them, indicating your degree of disagreement/agreement with them.

	**Strongly disagree**	**Quite disagree**	**Quite agree**	**Strongly agree**
I can get what I need in this community				
This community helps me fulfil my needs				
I feel like a member of this community				
I belong in this community				
I have a say about what goes on in my community				
People in this community are good at influencing each another				
I feel connected to this community				
I have a good bond with others in this community				

Imagine the Place where you live in the next five years. How will you live here?

**Much worse**	**Quite worse**	**Neither worse nor better**	**Quite better**	**Much better**

Session 2 – SOCIAL CONTEX

People around me (i.e., my family, my friends, acquaintances, colleagues):

	**Not at all**	**A little**	**Quite**	**Very**
I find comfort in them				
I get solidarity and the moral support I need from them				
I can share with them my problems and doubts				
They are willing to help me make decisions				
I can count on them when things go wrong				
I feel they are close to me				
I can share with them my joys and successes				
Take care of me				

Below are a series of statements. Please respond to all of them, indicating in what degree you agree/disagree with them

	Strongly disagree	Quite disagree	Quite agree	Strongly agree
There's little use in writing to public officials because often they aren't really interested in the problems of the average man				
Nowadays a person has to live pretty much for today and let tomorrow take care of itself				
In spite of what some people say, the lot of the average man is getting worse, not better				
It's hardly fair to bring children into the world, the way things look for the future				
These days a person doesn't really know whom he can count on				
Immigrants are a source of cultural enrichment				
Sometimes one has to break the rules to help one’s loved ones				
Those who succeed in the life has luck on their side				
People are unable to change				
It is useless to bustle, since you cannot affect what will be				
My life is determined by my own actions				
To a great extent, my life is controlled by accidental happenings				
My life is chiefly controlled by powerful others				
It is not possible at all to make any provision about the future				

Now you will find a list of words/phrases. Please choose <u>up to five</u> among them, the ones that in your opinion best express what you mean by *<u>wellbeing</u>* (If you like, further words may be added, in the lines “others”)

Safety	
Not being ill	
Fulfilment	
Health	
Capacity to love	
Detachment	
Adaptability	
Not suffering	
Other _________	

In your opinion, people's behaviour mainly depends on (choose only two options):

The temperament	
The emotions	
Economic interest	
The need to make sense of experience	
The predicted consequences of one's acts	
The need to defend one's reputation	
Norms and laws	
Shared values	
The feeling of group membership	

In your opinion, to succeed in life, how important is:

	Not at all	A little	Quite	Very
Understanding the world				
Acquiring knowledge				
Adjusting to the main trends				
Forming alliances with stronger people				
Having a few scruples				
Following rules				
Sharing				

Think of the coming years. Future will be

Far worse	A little worse	A little better	Far better

LASTLY, PLEASE GIVE US SOME DATA ABOUT YOURSELF

In comparison to a couple of years ago, considering your overall condition, your current life is.

Much worse	Quite worse	Neither worse nor better	Quite better	Much Better

In comparison to people of a similar age to you, your current condition of health is

Very bad	Bad	On average	Good	Very good

Where were you born?

Nation/Country ______________________

State/Region ______________________

City/Town ______________________

How many years have you been living in the Place where you live currently?

Less than 1 year	
1-4 years	
5-10 years	
11-20 years	
More than 20 years	

Indicate your status below

	Yes	No
Married or cohabitee		
Separated or divorced		
Widowed		
Living with family of origin		
Parent of one or more children		

How many people make up your current family nucleus? ____________

Up to now, your formal education (considering all levels, including higher education) has lasted

Less than 5 years	
6-9 years	
10-13 years	
14-17 years	
More than 17 years	

In which of the following categories does your main work activity fall into?

Managers and associate functions	(e.g. business services and administration managers; education managers; legislators and senior officials, heads of village)
Health associate professionals	(e.g. medical doctors; veterinarians; nursing and midwifery professionals; medical and pathology laboratory technicians)
Teaching professionals	(e.g. primary and secondary school teachers; higher education teachers; vocational education teachers)
Legal, social, cultural and related professionals/technicians	(e.g. economists; sociologists; social work and counselling professionals; religious professionals, journalists; lawyers, librarians, artists; chefs; police inspectors and detectives)
Science and engineering associate professionals/technicians	(e.g. meteorologists, chemists, biologists, engineers, architects, physicists; draughtspersons)

Other professionals/technicians	
Clerical support workers	(e.g. secretaries; data entry clerks; travel consultants and clerks; bank tellers and related clerks; contact centre information clerks; accounting and bookkeeping clerks)
Service and sales workers	(e.g. waiters, bartenders, other personal service workers; salespersons; health care assistants; teachers' aides; security guards)
Skilled Agricultural, Forestry and Fishery workers	(e.g. field crop and vegetable growers; gardeners, horticultural and nursery growers; animal producers; forestry and related workers; fishery workers, hunters and trappers; gatherers)
Craft and related trades workers	(e.g. bricklayers; carpenters and joiners; metal moulders; machinery mechanics and repairers; handicraft workers; electrical and electronic trades workers; butchers, fishmongers; tailors)
Plant and machine operators assemblers	(e.g. miners; assemblers; heavy truck and bus drivers; taxi and van drivers)
Armed forces occupations	
Student	
Housewife	
Looking for first job	
Not currently engaged in employment	
Retired	
Other_____________________	

In your time free from work, are you engaging in activities and initiatives at the service of your community?

Yes □ **No** □

If yes, mainly of what kind?

Social and health care service	
Socio-cultural animation	
Civic and politic participation	
Environmental protection	

We have finished. Thanks for your collaboration!

Email to which updates on the survey and final report will be sent _____________
