# Supplementary material for: Symbolic universes between present and future of Europe. First results of the map of European societies' cultural milieu
Source: PLoS One. 2018 Jan 3;13(1):e0189885. doi: 10.1371/journal.pone.0189885 (PMC5752019; doi:10.1371/journal.pone.0189885)
Supplement: S1 Table — Description of the 3 main factorial dimensions. (DOCX) [file pone.0189885.s002.docx]

S1 Table. Multiple Correspondence Analysis. Description of the 3 main factorial dimensions

1. *Factorial dimension 1 (23.9% inertia^§^)**

| *N* | *Items* | *Modalities* | *Coordinates* |
| --- | --- | --- | --- |
| F1.1 | AGREEMENT/DISAGREEMENT.-A person doesn't really know whom he can count on | strongly agree | -15.77 |
| F1.2 | AGREEMENT/DISAGREEMENT- The lot of the average man is getting worse | strongly agree | -14.50 |
| F1.3 | FUTURE WILL BE- | far worse | -14.45 |
| F1.4 | AGREEMENT/DISAGREEMENT-It is not possible at all to make any provision | strongly agree | -14.15 |
| F1.5 | AGREEMENT/DISAGREEMENT-It is useless to bustle. since you cannot affect | strongly agree | -13.39 |
| F1.6 | AGREEMENT/DISAGREEMENT-My life is chiefly controlled by powerful others | strongly agree | -12.56 |
| F1.7 | AGREEMENT/DISAGREEMENT-People are unable to change | strongly agree | -12.34 |
| F1.8 | AGREEMENT/DISAGREEMENT-There's little use in writing to public officials | strongly agree | -12.33 |
| F1.9 | AGREEMENT/DISAGREEMENT-It's hardly fair to bring children into the world | strongly agree | -12.33 |
| F1.10 | RELIABILITY-Public Administration | not at all | -11.09 |
| F1.11 | AGREEMENT/DISAGREEMENT-Those who succeed in the life has luck on their side | strongly agree | -10.92 |
| F1.12 | HOW YOU WILL LIVE IN THE PLACE YOU LIVE IN NEXT 5 Y- | much worse | -10.65 |
| F1.13 | RELIABILITY-Police | not at all | -10.63 |
| F1.14 | AGREEMENT/DISAGREEMENT-Sometimes to break the rules to help one’s loved | strongly agree | -10.60 |
| F1.15 | AGREEMENT/DISAGREEMENT-Nowadays a person has to live pretty much for today | strongly agree | -10.01 |
| F1.16 | RELIABILITY-Health care services | not at all | -9.29 |
| F1.17 | AGREEMENT/DISAGREEMENT-My life is controlled by accidental happenings | strongly agree | -9.04 |
| F1.18 | AGREEMENT/DISAGREEMENT-Immigrants are a source of cultural enrichment | strongly disagree | -8.80 |
| F1.19 | RELIABILITY-Companies | not at all | -8.69 |
| F1.20 | TO SUCCEED IN LIFE-Forming alliances with stronger people | very | -8.63 |
| F1.21 | CURRENT LIFE | Quite worse | -7.99 |
| F1.22 | CURRENT LIFE | Much worse | -7.44 |
| F1.23 | RELIABILITY-Schools | not at all | -7 |
| F1.24 | WELLBEING IS-Not suffering | Yes | -6.79 |
| F1.25 | TO SUCCEED IN LIFE-Having a few scruples | very | -6.16 |
| F1.26 | WELLBEING IS-Not being ill | Yes | -6.06 |
| F1.27 | RELIABILITY-Schools | not very | -5.97 |
| F1.28 | WELLBEING IS-Fulfilment | No | -5.79 |
| F1.29 | TO SUCCEED IN LIFE-Acquiring knowledge | not very | -5.22 |
| F1.30 | AGREEMENT/DISAGREEMENT-My life is determined by my own actions | strongly agree | -5.13 |
| F1.31 | TO SUCCEED IN LIFE-Following rules | not at all | -5.10 |
| F1.32 | BEHAVIUR DEPENDS ON-Economic interest | Yes | -4.90 |
| F1.33 | TO SUCCEED IN LIFE-Sharing | not at all | -4.84 |
| F1.34 | TO SUCCEED IN LIFE-Adjusting to the main trends | very | -4.70 |
| F1.35 | TO SUCCEED IN LIFE-Following rules | very | -4.66 |
| F1.36 | AGREEMENT/DISAGREEMENT-My life is determined by my own actions | strongly disagree | -4.49 |
| F1.37 | RELIABILITY-Health care services | not very | -4.46 |
| F1.38 | RELIABILITY-Police | not very | -4.01 |
| F1.39 | HOW YOU WILL LIVE IN THE PLACE YOU LIVE IN NEXT 5 Y- | quite worse | -3.94 |
| F1.40 | TO SUCCEED IN LIFE-Acquiring knowledge | not at all | -3.81 |
| F1.41 | AGREEMENT/DISAGREEMENT-My life is determined by my own actions | quite disagree | -3.70 |
| F1.42 | AGREEMENT/DISAGREEMENT-My life is controlled by accidental happenings | quite agree | -3.40 |
| F1.43 | WELLBEING IS-Adaptability | No | -3.31 |
| F1.44 | TO SUCCEED IN LIFE-Understanding the world | not very | -3.29 |
| F1.45 | BEHAVIUR DEPENDS ON-Shared values | No | -3.18 |
| *CENTRAL ZONE* | | | |
| F1.46 | BEHAVIUR DEPENDS ON-Shared values | Yes | 3.09 |
| F1.47 | WELLBEING IS-Adaptability | Yes | 3.24 |
| F1.48 | TO SUCCEED IN LIFE-Acquiring knowledge | quite | 3.34 |
| F1.49 | AGREEMENT/DISAGREEMENT-It's hardly fair to bring children into the world | strongly disagree | 3.50 |
| F1.50 | AGREEMENT/DISAGREEMENT- The lot of the average man is getting worse | quite agree | 4 |
| F1.51 | TO SUCCEED IN LIFE-Understanding the world | quite | 4.02 |
| F1.52 | TO SUCCEED IN LIFE-Forming alliances with stronger people | not very | 4.11 |
| F1.53 | TO SUCCEED IN LIFE-Having a few scruples | not very | 4.12 |
| F1.54 | TO SUCCEED IN LIFE-Forming alliances with stronger people | quite | 4.14 |
| F1.55 | RELIABILITY-Schools | quite | 4.16 |
| F1.56 | TO SUCCEED IN LIFE-Adjusting to the main trends | quite | 4.37 |
| F1.57 | AGREEMENT/DISAGREEMENT-It is not possible at all to make any provision | strongly disagree | 4.48 |
| F1.58 | AGREEMENT/DISAGREEMENT-It's hardly fair to bring children into the world | quite disagree | 4.62 |
| F1.59 | RELIABILITY-Health care services | quite | 4.66 |
| F1.60 | AGREEMENT/DISAGREEMENT-Sometimes to break the rules to help one’s loved | quite disagree | 4.79 |
| F1.61 | BEHAVIUR DEPENDS ON-Economic interest | No | 4.82 |
| F1.62 | RELIABILITY-Health care services | very | 4.87 |
| F1.63 | HOW YOU WILL LIVE IN THE PLACE YOU LIVE IN NEXT 5 Y- | quite better | 5.10 |
| F1.64 | TO SUCCEED IN LIFE-Sharing | quite | 5.10 |
| F1.65 | AGREEMENT/DISAGREEMENT-There's little use in writing to public officials | quite agree | 5.19 |
| F1.66 | AGREEMENT/DISAGREEMENT-Sometimes to break the rules to help one’s loved | quite agree | 5.22 |
| F1.67 | AGREEMENT/DISAGREEMENT-Those who succeed in the life has luck on their side | quite disagree | 5.56 |
| F1.68 | AGREEMENT/DISAGREEMENT-Immigrants are a source of cultural enrichment | quite agree | 5.69 |
| F1.69 | WELLBEING IS-Fulfilment | Yes | 5.69 |
| F1.70 | TO SUCCEED IN LIFE-Following rules | quite | 5.84 |
| F1.71 | RELIABILITY-Companies | quite | 5.88 |
| F1.72 | WELLBEING IS-Not being ill | No | 6.02 |
| F1.73 | AGREEMENT/DISAGREEMENT-People are unable to change | quite disagree | 6.05 |
| F1.74 | AGREEMENT/DISAGREEMENT-There's little use in writing to public officials | quite disagree | 6.28 |
| F1.75 | CURRENT LIFE | Quite better | 6.54 |
| F1.76 | AGREEMENT/DISAGREEMENT-My life is controlled by accidental happenings | quite disagree | 6.73 |
| F1.77 | WELLBEING IS-Not suffering | No | 6.73 |
| F1.78 | RELIABILITY-Police | quite | 7.01 |
| F1.79 | RELIABILITY-Public Administration | quite | 7.08 |
| F1.80 | AGREEMENT/DISAGREEMENT-My life is chiefly controlled by powerful others | quite disagree | 7.25 |
| F1.81 | AGREEMENT/DISAGREEMENT-Nowadays a person has to live pretty much for today | quite disagree | 7.38 |
| F1.82 | AGREEMENT/DISAGREEMENT-My life is determined by my own actions | quite agree | 7.95 |
| F1.83 | AGREEMENT/DISAGREEMENT-It is useless to bustle. since you cannot affect | quite disagree | 8.08 |
| F1.84 | AGREEMENT/DISAGREEMENT- The lot of the average man is getting worse | quite disagree | 8.82 |
| F1.85 | AGREEMENT/DISAGREEMENT-It is not possible at all to make any provision | quite disagree | 8.84 |
| F1.86 | AGREEMENT/DISAGREEMENT-A person doesn't really know whom he can count on | quite disagree | 8.96 |
| F1.87 | FUTURE WILL BE- | a little better | 9.07 |

*Reported items with coordinate >|3|

1. Factorial dimension 2 (9.1% inertia^§^)*

| *N* | *Items* | *Modalities* | *Coordinates* |
| --- | --- | --- | --- |
| F2.1 | AGREEMENT/DISAGREEMENT-It is useless to bustle, since you cannot affect | strongly disagree | -10.83 |
| F2.2 | RELIABILITY-Health care services | very | -9.37 |
| F2.3 | TO SUCCEED IN LIFE-Following rules | very | -9.37 |
| F2.4 | AGREEMENT/DISAGREEMENT-My life is chiefly controlled by powerful others | strongly disagree | -8.93 |
| F2.5 | AGREEMENT/DISAGREEMENT- The lot of the average man is getting worse | strongly disagree | -8.73 |
| F2.6 | RELIABILITY-Police | very | -8.70 |
| F2.7 | RELIABILITY-Companies | very | -8.61 |
| F2.8 | AGREEMENT/DISAGREEMENT-A person doesn't really know whom he can count on | strongly disagree | -8.60 |
| F2.9 | AGREEMENT/DISAGREEMENT-Sometimes to break the rules to help one’s loved | strongly disagree | -8.57 |
| F2.10 | AGREEMENT/DISAGREEMENT-People are unable to change | strongly disagree | -8.55 |
| F2.11 | TO SUCCEED IN LIFE-Sharing | very | -8.55 |
| F2.12 | RELIABILITY-Public transport | very | -8.40 |
| F2.13 | AGREEMENT/DISAGREEMENT-My life is determined by my own actions | strongly agree | -8.36 |
| F2.14 | AGREEMENT/DISAGREEMENT-It's hardly fair to bring children into the world | strongly disagree | -8.23 |
| F2.15 | AGREEMENT/DISAGREEMENT-It is not possible at all to make any provision | strongly disagree | -8.04 |
| F2.16 | AGREEMENT/DISAGREEMENT-My life is controlled by accidental happenings | strongly disagree | -7.98 |
| F2.17 | RELIABILITY-Public Administration | very | -7.88 |
| F2.18 | RELIABILITY-Schools | very | -7.48 |
| F2.19 | AGREEMENT/DISAGREEMENT-Those who succeed in the life has luck on their side | strongly disagree | -7.46 |
| F2.20 | AGREEMENT/DISAGREEMENT-There's little use in writing to public officials | strongly disagree | -7.33 |
| F2.21 | TO SUCCEED IN LIFE-Acquiring knowledge | very | -7.27 |
| F2.22 | FUTURE WILL BE- | far better | -7.01 |
| F2.23 | TO SUCCEED IN LIFE-Understanding the world | very | -6.65 |
| F2.24 | TO SUCCEED IN LIFE-Forming alliances with stronger people | not at all | -6.64 |
| F2.25 | CURRENT LIFE | Much better | -6.34 |
| F2.26 | TO SUCCEED IN LIFE-Having a few scruples | not at all | -5.35 |
| F2.27 | AGREEMENT/DISAGREEMENT-Nowadays a person has to live pretty much for today | strongly disagree | -5.26 |
| F2.28 | TO SUCCEED IN LIFE-Adjusting to the main trends | not at all | -5.15 |
| F2.29 | HOW YOU WILL LIVE IN THE PLACE YOU LIVE IN NEXT 5 Y- | much better | -5.02 |
| F2.30 | AGREEMENT/DISAGREEMENT-My life is controlled by accidental happenings | strongly agree | -4.75 |
| F2.31 | AGREEMENT/DISAGREEMENT-Immigrants are a source of cultural enrichment | strongly agree | -4.32 |
| F2.32 | AGREEMENT/DISAGREEMENT- The lot of the average man is getting worse | quite disagree | -4.28 |
| F2.33 | TO SUCCEED IN LIFE-Adjusting to the main trends | very | -3.83 |
| F2.34 | AGREEMENT/DISAGREEMENT-There's little use in writing to public officials | quite disagree | -3.77 |
| F2.35 | BEHAVIUR DEPENDS ON-Shared values | Yes | -3.66 |
| F2.36 | TO SUCCEED IN LIFE-Having a few scruples | very | -3.65 |
| F2.38 | BEHAVIUR DEPENDS ON-Economic interest | No | -3.34 |
| F2.39 | RELIABILITY-Public Administration | quite | -3.12 |
| F2.40 | AGREEMENT/DISAGREEMENT-A person doesn't really know whom he can count on | quite disagree | -3.09 |
|  | *C E N T R A L ZONE* | | |
| F2.41 | BEHAVIUR DEPENDS ON-The need to defend one’s reputation | Yes | 3.01 |
| F2.42 | TO SUCCEED IN LIFE-Sharing | not very | 3.39 |
| F2.43 | BEHAVIUR DEPENDS ON-Economic interest | Yes | 3.48 |
| F2.44 | RELIABILITY-Public transport | quite | 3.49 |
| F2.45 | TO SUCCEED IN LIFE-Adjusting to the main trends | quite | 3.55 |
| F2.46 | AGREEMENT/DISAGREEMENT-It's hardly fair to bring children into the world | quite disagree | 3.57 |
| F2.47 | AGREEMENT/DISAGREEMENT-My life is determined by my own actions | quite disagree | 3.75 |
| F2.48 | TO SUCCEED IN LIFE-Having a few scruples | quite | 3.79 |
| F2.49 | BEHAVIUR DEPENDS ON-Shared values | No | 3.81 |
| F2.50 | TO SUCCEED IN LIFE-Following rules | quite | 4.02 |
| F2.51 | RELIABILITY-Health care services | not very | 4.13 |
| F2.52 | TO SUCCEED IN LIFE-Having a few scruples | not very | 4.18 |
| F2.53 | RELIABILITY-Police | not very | 4.25 |
| F2.54 | HOW YOU WILL LIVE IN THE PLACE YOU LIVE IN NEXT 5 Y- | quite worse | 4.34 |
| F2.55 | RELIABILITY-Schools | not very | 4.37 |
| F2.56 | TO SUCCEED IN LIFE-Acquiring knowledge | not very | 4.44 |
| F2.57 | RELIABILITY-Companies | not very | 4.45 |
| F2.58 | TO SUCCEED IN LIFE-Following rules | not very | 4.62 |
| F2.59 | AGREEMENT/DISAGREEMENT-My life is controlled by accidental happenings | quite agree | 4.96 |
| F2.60 | TO SUCCEED IN LIFE-Sharing | quite | 5.02 |
| F2.61 | AGREEMENT/DISAGREEMENT-Nowadays a person has to live pretty much for today | quite agree | 5.28 |
| F2.62 | TO SUCCEED IN LIFE-Understanding the world | quite | 5.51 |
| F2.63 | AGREEMENT/DISAGREEMENT-It is not possible at all to make any provision | quite agree | 5.54 |
| F2.64 | AGREEMENT/DISAGREEMENT-My life is determined by my own actions | quite agree | 5.72 |
| F2.65 | TO SUCCEED IN LIFE-Acquiring knowledge | quite | 6.03 |
| F2.66 | AGREEMENT/DISAGREEMENT-It's hardly fair to bring children into the world | quite agree | 6.27 |
| F2.67 | AGREEMENT/DISAGREEMENT-My life is chiefly controlled by powerful others | quite agree | 6.35 |
| F2.68 | AGREEMENT/DISAGREEMENT-People are unable to change | quite agree | 6.43 |
| F2.69 | AGREEMENT/DISAGREEMENT-Sometimes to break the rules to help one’s loved | quite agree | 6.44 |
| F2.70 | AGREEMENT/DISAGREEMENT-Those who succeed in the life has luck on their side | quite agree | 6.50 |
| F2.71 | RELIABILITY-Public Administration | not very | 6.50 |
| F2.72 | FUTURE WILL BE- | a little worse | 6.72 |
| F2.73 | AGREEMENT/DISAGREEMENT-There's little use in writing to public officials | quite agree | 6.83 |
| F2.74 | AGREEMENT/DISAGREEMENT- The lot of the average man is getting worse | quite agree | 7.27 |
| F2.75 | AGREEMENT/DISAGREEMENT-A person doesn't really know whom he can count on | quite agree | 8.94 |
| F2.76 | AGREEMENT/DISAGREEMENT-It is useless to bustle. since you cannot affect | quite agree | 9.61 |

1. Factorial dimension 3 (4.7% inertia^§^)*

| *N* | *Items* | *Modalities* | *Coordinates* |
| --- | --- | --- | --- |
| F3.1 | RELIABILITY-Schools | very | -9.60 |
| F3.2 | RELIABILITY-Public Administration | very | -9.54 |
| F3.3 | RELIABILITY-Police | very | -8.87 |
| F3.4 | WELLBEING IS-Not being ill | Yes | -8.07 |
| F3.5 | RELIABILITY-Companies | very | -7.49 |
| F3.6 | AGREEMENT/DISAGREEMENT-Those who succeed in the life has luck on their side | strongly agree | -7.28 |
| F3.7 | TO SUCCEED IN LIFE-Forming alliances with stronger people | very | -7 |
| F3.8 | RELIABILITY-Health care services | very | -6.52 |
| F3.9 | TO SUCCEED IN LIFE-Adjusting to the main trends | quite | -6.33 |
| F3.10 | WELLBEING IS-Not suffering | Yes | -6.16 |
| F3.11 | AGREEMENT/DISAGREEMENT-My life is controlled by accidental happenings | strongly agree | -5.80 |
| F3.12 | RELIABILITY-Public transport | very | -5.68 |
| F3.13 | RELIABILITY-Public Administration | quite | -5.68 |
| F3.14 | AGREEMENT/DISAGREEMENT-People are unable to change | quite agree | -5.51 |
| F3.15 | TO SUCCEED IN LIFE-Adjusting to the main trends | very | -4.73 |
| F3.16 | TO SUCCEED IN LIFE-Having a few scruples | quite | -4.66 |
| F3.17 | AGREEMENT/DISAGREEMENT-People are unable to change | strongly agree | -4.64 |
| F3.18 | TO SUCCEED IN LIFE-Following rules | very | -4.32 |
| F3.19 | WELLBEING IS-Fulfilment | No | -4.25 |
| F3.20 | AGREEMENT/DISAGREEMENT-Those who succeed in the life has luck on their side | quite agree | -4.19 |
| F3.21 | AGREEMENT/DISAGREEMENT-My life is controlled by accidental happenings | quite agree | -3.70 |
| F3.22 | RELIABILITY-Companies | quite | -3.68 |
| F3.23 | AGREEMENT/DISAGREEMENT-Immigrants are a source of cultural enrichment | quite disagree | -3.67 |
| F3.24 | AGREEMENT/DISAGREEMENT- The lot of the average man is getting worse | quite agree | -3.58 |
| F3.25 | WELLBEING IS-Capacity to love | No | -3.44 |
| F3.26 | AGREEMENT/DISAGREEMENT-It is useless to bustle, since you cannot affect | strongly agree | -3.33 |
| F3.27 | CURRENT LIFE | Neither worse nor be | -3.31 |
| F3.28 | WELLBEING IS-Safety | Yes | -3.23 |
| F3.29 | AGREEMENT/DISAGREEMENT-It's hardly fair to bring children into the world | strongly agree | -3.16 |
| F3.30 | TO SUCCEED IN LIFE-Sharing | not very | -3.10 |
| F3.31 | AGREEMENT/DISAGREEMENT-A person doesn't really know whom he can count on | quite agree | -3.10 |
| *C E N T R A L ZONE* | | | |
| F3.32 | BEHAVIUR DEPENDS ON-Economic interest | No | 3.03 |
| F3.33 | TO SUCCEED IN LIFE-Following rules | not at all | 3.26 |
| F3.34 | TO SUCCEED IN LIFE-Forming alliances with stronger people | not very | 3.27 |
| F3.35 | AGREEMENT/DISAGREEMENT-It is not possible at all to make any provision | strongly disagree | 3.35 |
| F3.36 | WELLBEING IS-Safety | No | 3.41 |
| F3.37 | WELLBEING IS-Capacity to love | Yes | 3.58 |
| F3.38 | AGREEMENT/DISAGREEMENT-A person doesn't really know whom he can count on | quite disagree | 3.67 |
| F3.39 | RELIABILITY-Health care services | not very | 3.83 |
| F3.40 | AGREEMENT/DISAGREEMENT-My life is chiefly controlled by powerful others | strongly disagree | 4 |
| F3.41 | RELIABILITY-Police | not at all | 4.33 |
| F3.42 | WELLBEING IS-Fulfilment | Yes | 4.38 |
| F3.43 | RELIABILITY-Public transport | not very | 4.46 |
| F3.44 | AGREEMENT/DISAGREEMENT-Immigrants are a source of cultural enrichment | strongly agree | 4.48 |
| F3.45 | AGREEMENT/DISAGREEMENT-Those who succeed in the life has luck on their side | quite disagree | 4.72 |
| F3.46 | RELIABILITY-Companies | not at all | 4.73 |
| F3.47 | RELIABILITY-Public transport | not at all | 5.12 |
| F3.48 | RELIABILITY-Public Administration | not at all | 5.25 |
| F3.49 | RELIABILITY-Health care services | not at all | 5.37 |
| F3.50 | RELIABILITY-Companies | not very | 5.54 |
| F3.51 | TO SUCCEED IN LIFE-Adjusting to the main trends | not very | 6.02 |
| F3.52 | RELIABILITY-Police | not very | 6.15 |
| F3.53 | WELLBEING IS-Not suffering | No | 6.31 |
| F3.54 | RELIABILITY-Schools | not very | 6.82 |
| F3.55 | TO SUCCEED IN LIFE-Having a few scruples | not at all | 6.92 |
| F3.56 | TO SUCCEED IN LIFE-Forming alliances with stronger people | not at all | 6.98 |
| F3.57 | AGREEMENT/DISAGREEMENT-Those who succeed in the life has luck on their side | strongly disagree | 7 |
| F3.58 | AGREEMENT/DISAGREEMENT-It is useless to bustle, since you cannot affect | strongly disagree | 7.23 |
| F3.59 | TO SUCCEED IN LIFE-Adjusting to the main trends | not at all | 7.49 |
| F3.60 | RELIABILITY-Public Administration | not very | 7.53 |
| F3.61 | AGREEMENT/DISAGREEMENT-My life is controlled by accidental happenings | strongly disagree | 8.09 |
| F3.62 | WELLBEING IS-Not being ill | No | 8.23 |
| F3.63 | AGREEMENT/DISAGREEMENT-People are unable to change | strongly disagree | 9.56 |

§ calculated in accordance to the Benzecri’s simplified formula of revaluation

*Reported items with coordinate >|3|
