# Supplementary material for: Symbolic universes between present and future of Europe. First results of the map of European societies' cultural milieu
Source: PLoS One. 2018 Jan 3;13(1):e0189885. doi: 10.1371/journal.pone.0189885 (PMC5752019; doi:10.1371/journal.pone.0189885)
Supplement: S1 Fig — Factor 1 vs. Factor 2. (PDF) [file pone.0189885.s003.pdf]

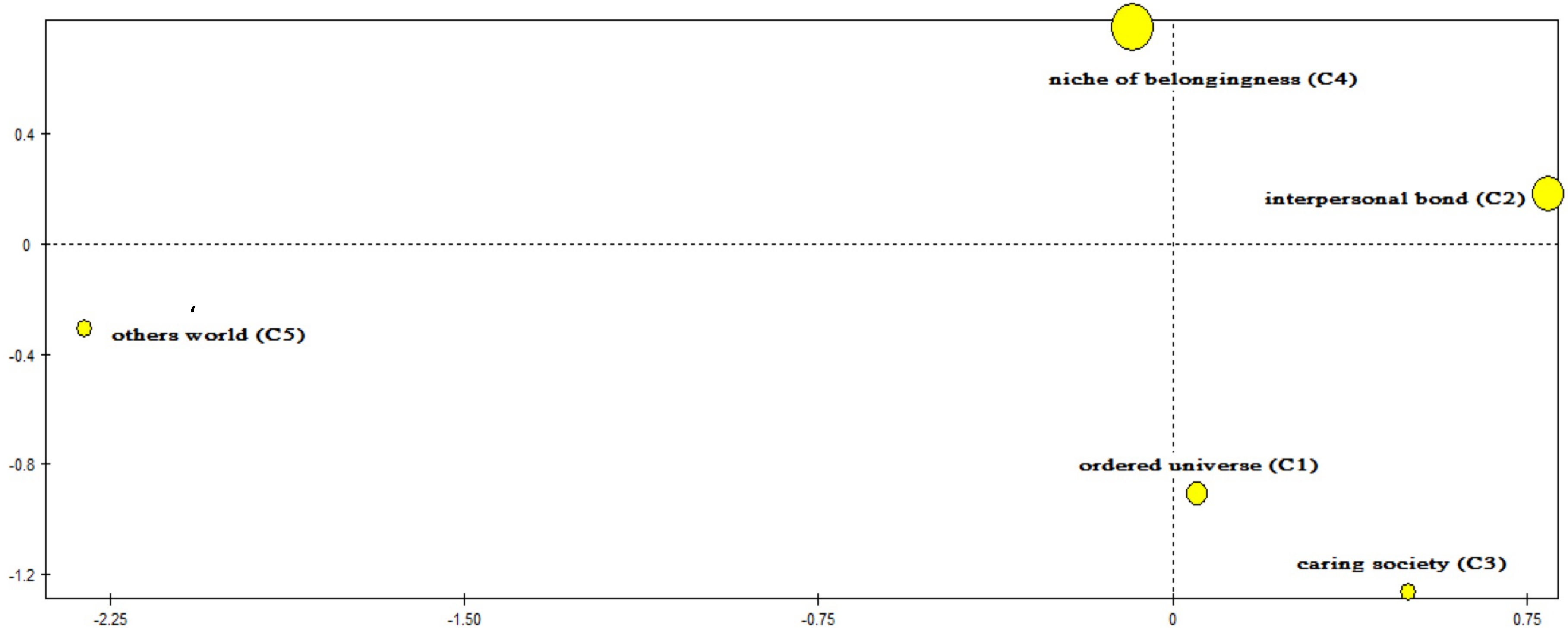

S1 Fig. Position of the symbolic universes on the MCA main factorial dimensions. Factor 1 vs. Factor 2

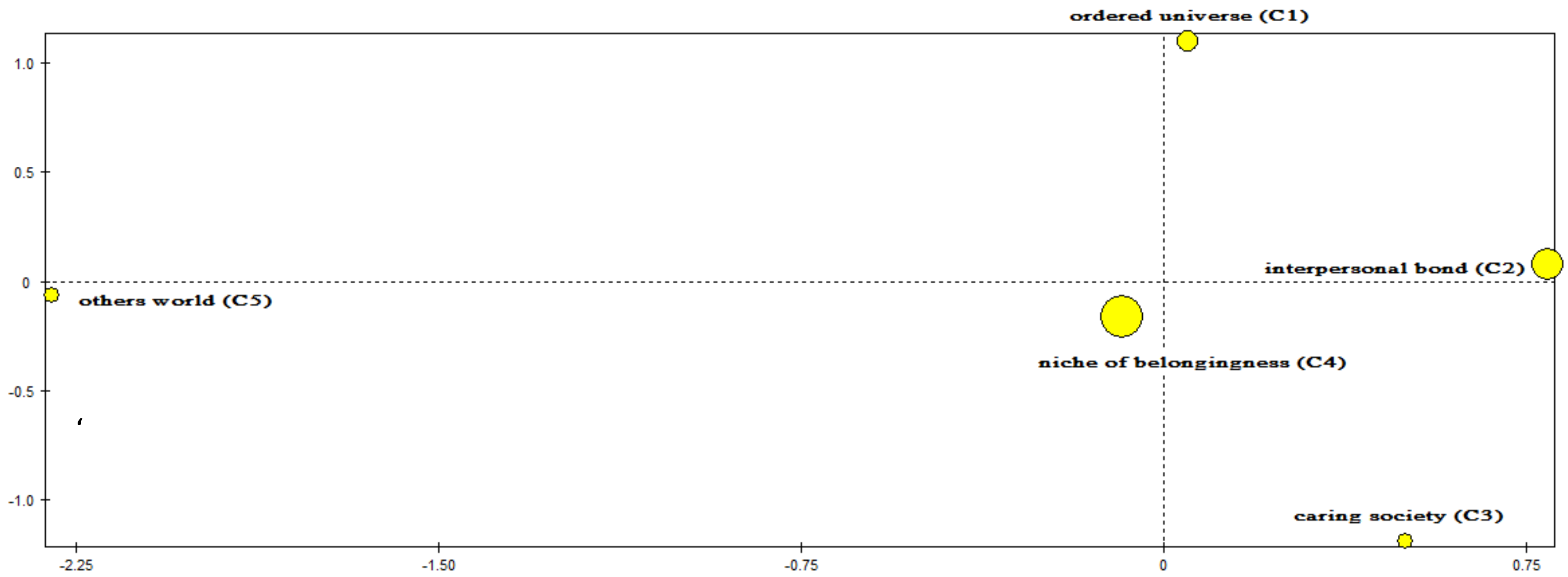

S2 Fig. Position of the symbolic universes on the MCA main factorial dimensions. Factor 1 vs. Factor 3
